# NIGT1 family proteins exhibit dual mode DNA recognition to regulate nutrient response-associated genes in Arabidopsis

**DOI:** 10.1371/journal.pgen.1009197

**Published:** 2020-11-02

**Authors:** Yoshiaki Ueda, Shohei Nosaki, Yasuhito Sakuraba, Takuya Miyakawa, Takatoshi Kiba, Masaru Tanokura, Shuichi Yanagisawa

**Affiliations:** 1 Biotechnology Research Center, The University of Tokyo, Bunkyo-ku, Tokyo, Japan; 2 Crop, Livestock and Environment Division, Japan International Research Center for Agricultural Sciences, Tsukuba, Ibaraki, Japan; 3 Graduate School of Agricultural and Life Sciences, The University of Tokyo, Bunkyo-ku, Tokyo, Japan; 4 Graduate School of Bioagricultural Sciences, Nagoya University, Chikusa, Nagoya, Japan; 5 Center for Sustainable Resource Science, RIKEN, Tsurumi, Yokohama, Japan; Peking University, CHINA

## Abstract

Fine-tuning of nutrient uptake and response is indispensable for maintenance of nutrient homeostasis in plants, but the details of underlying mechanisms remain to be elucidated. NITRATE-INDUCIBLE GARP-TYPE TRANSCRIPTIONAL REPRESSOR 1 (NIGT1) family proteins are plant-specific transcriptional repressors that function as an important hub in the nutrient signaling network associated with the acquisition and use of nitrogen and phosphorus. Here, by yeast two-hybrid assays, bimolecular fluorescence complementation assays, and biochemical analysis with recombinant proteins, we show that Arabidopsis NIGT1 family proteins form a dimer via the interaction mediated by a coiled-coil domain (CCD) in their N-terminal regions. Electrophoretic mobility shift assays defined that the NIGT1 dimer binds to two different motifs, 5'-GAATATTC-3' and 5'-GATTC-N_38_-GAATC-3', in target gene promoters. Unlike the dimer of wild-type NIGT1 family proteins, a mutant variant that could not dimerize due to amino acid substitutions within the CCD had lower specificity and affinity to DNA, thereby losing the ability to precisely regulate the expression of target genes. Thus, expressing the wild-type and mutant NIGT1 proteins in the *nigt1* quadruple mutant differently modified NIGT1-regulated gene expression and responses towards nitrate and phosphate. These results suggest that the CCD-mediated dimerization confers dual mode DNA recognition to NIGT1 family proteins, which is necessary to make proper controls of their target genes and nutrient responses. Intriguingly, two 5'-GATTC-3' sequences are present in face-to-face orientation within the 5'-GATTC-N_38_-GAATC-3' sequence or its complementary one, while two 5'-ATTC-3' sequences are present in back-to-back orientation within the 5'-GAATATTC-3' or its complementary one. This finding suggests a unique mode of DNA binding by NIGT1 family proteins and may provide a hint as to why target sequences for some transcription factors cannot be clearly determined.

## Author summary

Expression of genes is tightly regulated by transcription factors that physically interact with DNA. Transcription factors recognize specific DNA sequences and selectively regulate the expression of target genes through the specific interactions. We previously found that a group of plant-specific transcription factors, NIGT1 family proteins, recognize two different types of DNA sequences to modulate the expression of a number of genes associated with uptake of nitrogen and phosphorus or related metabolism in Arabidopsis. However, it was unknown how NIGT1 family proteins recognize different types of sequences. In this study, we reveal that dimerization of NIGT1 family proteins is essential to the dual specificity of NIGT1 family proteins and therefore loss of the dimerization ability alters specificity and affinity of NIGT1 family proteins to target DNA sequences. We further suggest that the dimerization of NIGT1 family proteins is an evolutionarily conserved feature to properly respond to changes in nutrient availability. These findings would advance our knowledge on the mode of DNA recognition by transcription factors and have further implications for structural basis on the diverse patterns of the interaction between DNA and transcription factors.

## Introduction

Plants are continuously exposed to fluctuations in soil nutrient levels because of changes in climatic conditions, microbial activities, and water flow [1–3]. To maintain nutrient homeostasis and ensure successful reproduction, plants employ a wide variety of responses that modulate nutrient uptake and metabolism. Response triggered by the shortage of phosphorus (P), one of the major macronutrients needed for plant growth, is one such response [4,5]. Under P-deficient conditions, plants increase the uptake of inorganic phosphate (Pi), the plant-accessible form of P, to maintain internal P concentration [5,6]. In addition, many plant species accumulate foliar anthocyanins under Pi-deficient conditions to effectively dissipate excess light energy and mitigate oxidative stress [5,6]. These responses, collectively termed as Pi starvation responses (PSRs), are induced by a Pi-dependent transcriptional regulation. Similarly, shortage of nitrogen (N), another macronutrient required in large amounts, also triggers a nutrient response. N shortage causes chlorosis and decreases photosynthesis and plant growth [7,8]. Most of the terrestrial plants uptake N in the form of nitrate [9], and respond to nitrate supply as well as long-term depletion of N via transcriptional reprogramming [10,11].

In *Arabidopsis thaliana* and rice (*Oryza sativa* L.), some of the GOLDEN2/ARR-B/Psr1 (GARP)-type transcription factors (TFs), such as PHOSPHATE STARVATION RESPONSE 1 (PHR1) and its homologs, play a major role in the initiation of PSR [5,6,12–14]. Although *PHR1* gene expression is hardly responsive to fluctuations in Pi availability [6,14], Pi-dependent interaction of PHR1 with SYG1/Pho81/XPR1 (SPX) domain-containing proteins (SPX proteins) modulates PHR1 activity. Under Pi-sufficient conditions, SPX proteins bind to PHR1, thus maintaining PHR1 in an inactive state [15–17]. However, upon Pi deprivation, the interaction between SPX proteins and PHR1 is weakened, and PHR1 is released from the PHR1–SPX complex; the released PHR1 then binds to the *cis*-element GNATATNC in target gene promoters, thus inducing their expression and initiating PSR [15,17–19]. On the other hand, the response to nitrate supply is orchestrated by NIN-LIKE PROTEIN (NLP) transcriptional activators in Arabidopsis and other plant species [11,20–22]. The NLP family proteins induce the expression of a range of N uptake or metabolism-related genes, such as *NRT2*.*1*, which encodes a high affinity nitrate transporter [22–25]. The NLP family proteins also regulate the expression of genes encoding other TFs, such as NITRATE-INDUCIBLE GARP-TYPE TRANSCRIPTIONAL REPRESSOR 1 (NIGT1) family proteins, to generate a complex regulatory network. Because the NIGT1 family proteins are GARP-type TFs involved in both down-regulation of nitrate supply-induced gene expression and repression of N starvation-inducible genes under N replete conditions, the NIGT1 family proteins play an indispensable and complex role in the transcriptional regulatory network in response to nitrate supply [24,26].

NIGT1 was first identified in rice as a transcriptional repressor, and overexpression of *OsNIGT1* altered N use [27]. Four Arabidopsis homologs of OsNIGT1, NIGT1.1-NIGT1.4, are similarly encoded by nitrate-inducible genes, and *NIGT1*.*4* is identical to *HYPERSENSITIVITY TO LOW PHOSPHATE-ELICITED PRIMARY ROOT SHORTENING 1* (*HRS1*) whose overexpression enhanced PSR in primary roots [28,29]. Recently, it was shown that NIGT1 family proteins directly bind to the promoters of *SPX1*, *SPX2*, and *SPX4* genes and promote PSRs, such as the accumulation of anthocyanins and expression of genes encoding Pi transporters (i.e., *PHT1*) and Pi uptake [30]. Intriguingly, in addition to the induction by NLP TFs upon nitrate supply, *NIGT1* genes are also induced by PHR1 under Pi-deficient conditions. Since NIGT1 family proteins suppress the expression of nitrate-inducible genes, such as *NRT2*.*1* and *NRT2*.*4*, and nitrate uptake [24,26], NIGT1 proteins play pivotal roles in modulating both nitrate-dependent Pi uptake and Pi-dependent nitrate uptake, which ultimately affect the stoichiometry of nitrate and Pi uptake [24,28,30,31].

NIGT1 family proteins demonstrate dual mode recognition of DNA sequence, i.e., these proteins bind to two types of *cis*-elements, GAATC (or its reverse complement GATTC) and GAATATTC [24,27,32], and the latter *cis*-element overlaps with the PHR1-binding sequence. While PHR1 forms a dimer through the coiled-coil domain (CCD) in its C-terminal region and recognizes palindromic sequences [14], some other GARP-type TFs in Arabidopsis (such as the ARABIDOPSIS RESPONSE REGULATOR10 [ARR10] and GOLDEN 2-LIKE1 [GLK1] and 2 [GLK2]) and rice (such as REGULATOR OF LEAF INCLINATION1 [RLI1]) bind to shorter non-palindromic sequences [33–35]. Considering that ARR10 binds to DNA as a monomer, as shown by X-ray crystallography [33], we proposed that NIGT1 family proteins bind to two types of *cis*-elements as a monomer as well as a dimer [32]. However, the molecular basis of dual mode DNA recognition by NIGT1 family proteins as the NIGT1–NIGT1 complex has not yet been confirmed. Furthermore, there is no evidence supporting the physiological importance of dual mode DNA recognition by NIGT1 family proteins.

Here, we show that the conserved N-terminal CCD of NIGT1 family proteins mediates NIGT1–NIGT1 interaction. We also show that this interaction enables NIGT1 family proteins to exist exclusively as dimers in solution. Consequently, authentic dual mode DNA recognition by NIGT1 family proteins involves their binding to two *cis*-elements, GAATATTC and GATTC-N_38_-GAATC, in target gene promoters. Interestingly, the sense and anti-sense strands of these elements include one GAAT motif; however, their directions in GAATATTC and GATTC-N_38_-GAATC sequences are opposite, predicting a unique mode of DNA-binding of NIGT1 family proteins. Furthermore, using wild-type NIGT1.1 and mutant NIGT1.1, we provide evidence supporting the physiological significance of the NIGT1 dimerization. Taken together, the results of this study enhance our understanding of the regulatory mechanisms underlying PSRs and nitrate responses in plants, and also provide important clues for resolving the complex *cis*-element recognition by TFs.

## Results

### Conserved N-terminal CCD mediates homomeric and heteromeric interactions among NIGT1 family proteins in yeast

**Fig 1:**
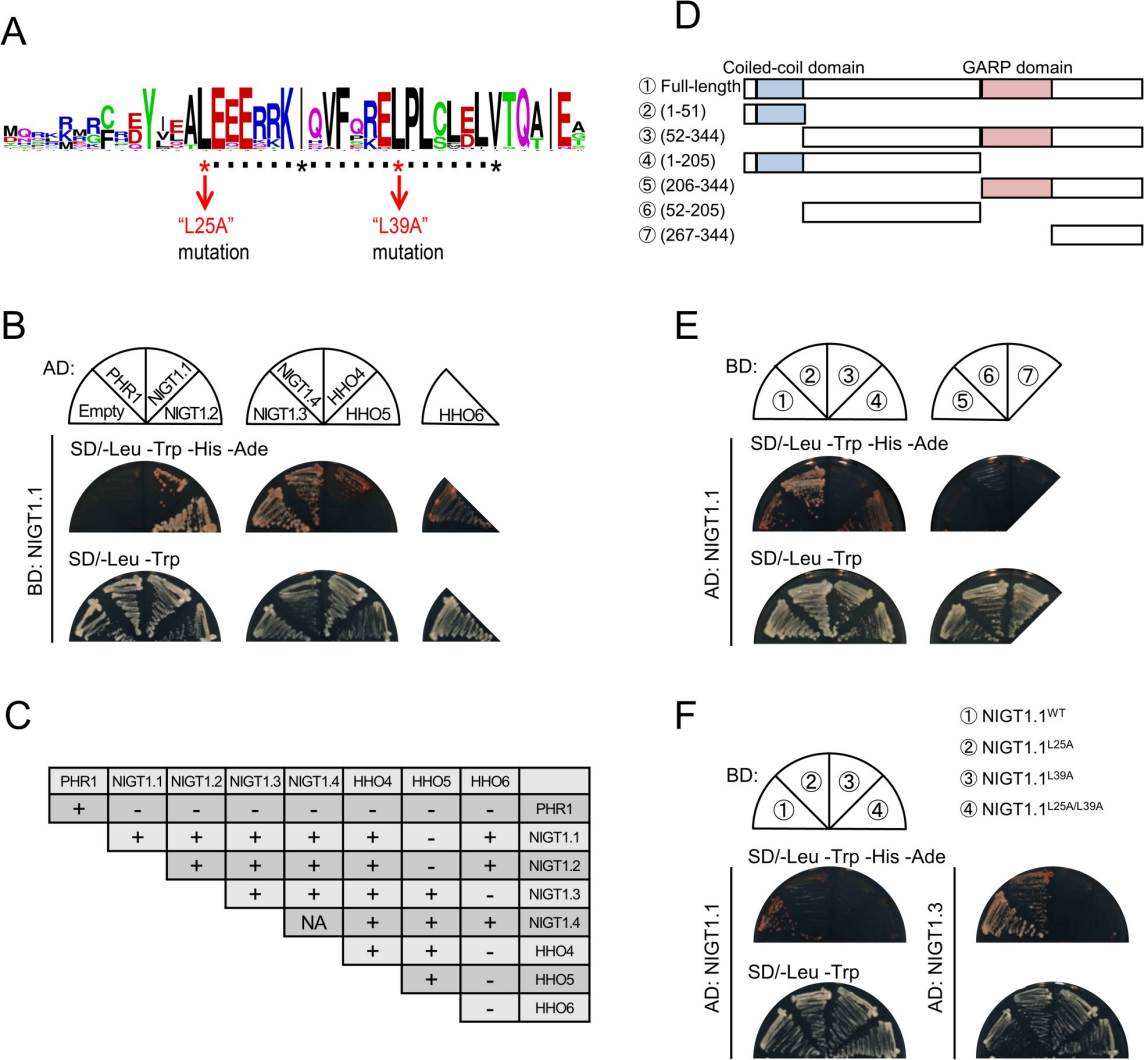
Amino acid sequence of the coiled-coil domain (CCD) of NIGT1 family proteins and its importance in protein–protein interactions in yeast. **(A)** Amino acid sequences of the CCDs of 22 NIGT1 family proteins from 13 plant species. Asterisks indicate conserved branched-chain amino acid residues (leucine [Leu], isoleucine [Ile], and valine [Val]) that appear after every seven residues. L25A and L39A indicate Leu to alanine [Ala] substitutions at amino acid positions 25 and 39, respectively. **(B)** Yeast two-hybrid (Y2H) assays using NIGT1.1 as the bait, and NIGT1 and HHO family proteins as preys. Full-length NIGT1 and HHO proteins were used for the assay, while truncated PHR1 carrying the GARP domain and CCD (208–362 aa) was used as a negative control. AD, GAL4 activation domain (prey); BD, GAL4 DNA-binding domain (bait). Synthetic defined (SD) medium lacking Leu and trytophan (Trp) (SD/-Leu/-Trp) or lacking Leu, Trp, histidine (His), and adenine (Ade) (SD/-Leu/-Trp/-His/-Ade) was used. **(C)** Interaction between NIGT1 and HHO family proteins in Y2H assays. “+” and “-” indicate the growth of colonies or no colonies, respectively, on SD/-Leu/-Trp/-His/-Ade. “NA” indicates that the combination was not examined since BD-NIGT1.4 activated transcription even in the absence of an interacting protein. Truncated PHR1 served as a negative control. See S2 Fig for all Y2H data. **(D)** Regions of NIGT1.1 used as bait in **(E)**. Numbers indicate N- and C-terminal amino acid residues in each region. Positions of the CCD (11–52 aa) and GARP DNA-binding domain (206–266 aa) are indicated. **(E)** Y2H assay using different regions of NIGT1.1 as the bait and full-length NIGT1.1 as the prey. **(F)** Y2H assay using mutant NIGT1.1 proteins. NIGT1.1^WT^, wild-type NIGT1.1; NIGT1.1^L25A^ and NIGT1.1^L39A^, mutant NIGT1.1 proteins carrying Leu to Ala substitution only at amino acid position 25 or 39, respectively; NIGT1.1^L25A/L39A^, mutant NIGT1.1 protein carrying two Leu to Ala substitutions at amino acid positions 25 and 39.

Approximately 50% of the GARP-type TFs carry a CCD, which potentially mediates protein–protein interactions [36]. Therefore, we conducted a protein domain search using NIGT1 family proteins and HRS1 HOMOLOG (HHO) family proteins, which are closely related to NIGT1 family proteins but encoded by nitrate-non-inducible genes. The results revealed a conserved amino acid sequence representing a coiled-coil motif in the N-terminal region of NIGT1 and HHO family proteins (S1 Fig). Amino acid sequence alignment of the predicted CCDs of 22 NIGT1 family proteins from 13 plant species reported previously [26] revealed that the domain includes heptad repeats of branched-chain amino acids (leucine [Leu], isoleucine [Ile], and valine [Val]) spanning 22 amino acid residues (Fig 1A). This structural feature resembles that of the Leu zipper domain [37], which is frequently involved in the dimerization of DNA-binding proteins. We, therefore, examined the protein–protein interactions by yeast two-hybrid (Y2H) assays using four NIGT1 family proteins (NIGT1.1–1.4) and three HHO family proteins (HHO4–6) from Arabidopsis. NIGT1.1 interacted with all four NIGT1 family proteins as well as with HHO4 and HHO6, but not with HHO5 (Fig 1B). Examination of all pairwise combinations of proteins revealed that the four NIGT1 family proteins and HHO4 interacted with each other; however, their interaction with HHO5 and HHO6 was observed only in certain combinations, suggesting that HHO5 and HHO6 perform distinct functions compared with NIGT1 family proteins and HHO4 (Fig 1C; S2 Fig). In this assay, truncated PHR1 carrying the GARP domain and CCD (208–362 amino acids [aa]) was used as a negative control, because full-length PHR1 forms a homo dimer via a CCD in its C-terminal region [14] and activates transcription in yeast [17]. PHR1 interacted with itself but did not interact with any of the NIGT1 and HHO family proteins, confirming the specificity of the NIGT1–NIGT1 interactions (Fig 1B and 1C; S2 Fig).

Next, to investigate whether the predicted CCD is necessary for NIGT1–NIGT1 interaction, we performed Y2H assays using deletion variants of NIGT1.1 (Fig 1D). The results indicated that the N-terminal region (1–51 aa) is required and sufficient for NIGT1–NIGT1 interaction, whereas other regions are not involved in this interaction (Fig 1E). Among the branched-chain amino acids comprising the heptad repeats in the CCD, two Leu residues (amino acid positions 25 and 39 in NIGT1.1) and an Ile residue (amino acid position 32 in NIGT1.1) were conserved among all examined NIGT1 proteins (Fig 1A). Positions of these amino acid residues correspond to those of hydrophobic amino acid residues that constitute the hydrophobic surface of the Leu zipper domain [37–39]. Thus, we examined whether the wild-type NIGT1.1 protein (NIGT1.1^WT^) interacts with mutant NIGT1.1 proteins in which the 25^th^ and/or 39^th^ Leu residue was substituted by the non-branched amino acid alanine (Ala) (NIGT1.1^L25A^, NIGT1.1^L39A^, and NIGT1.1^L25A/L39A^). The Y2H assays showed a drastic reduction in the NIGT1.1–NIGT1.1 interaction in these mutant NIGT1.1 proteins (Fig 1F). A similar impairment in protein–protein interaction was observed when NIGT1.3 was used as an interacting partner for the mutant NIGT1.1 proteins (Fig 1F). These results suggest that Leu residues in the N-terminal CCD are essential for homomeric and heteromeric interactions between NIGT1 family proteins.

### Mutations within the CCD abolish NIGT1–NIGT1 interaction *in planta*

**Fig 2:**
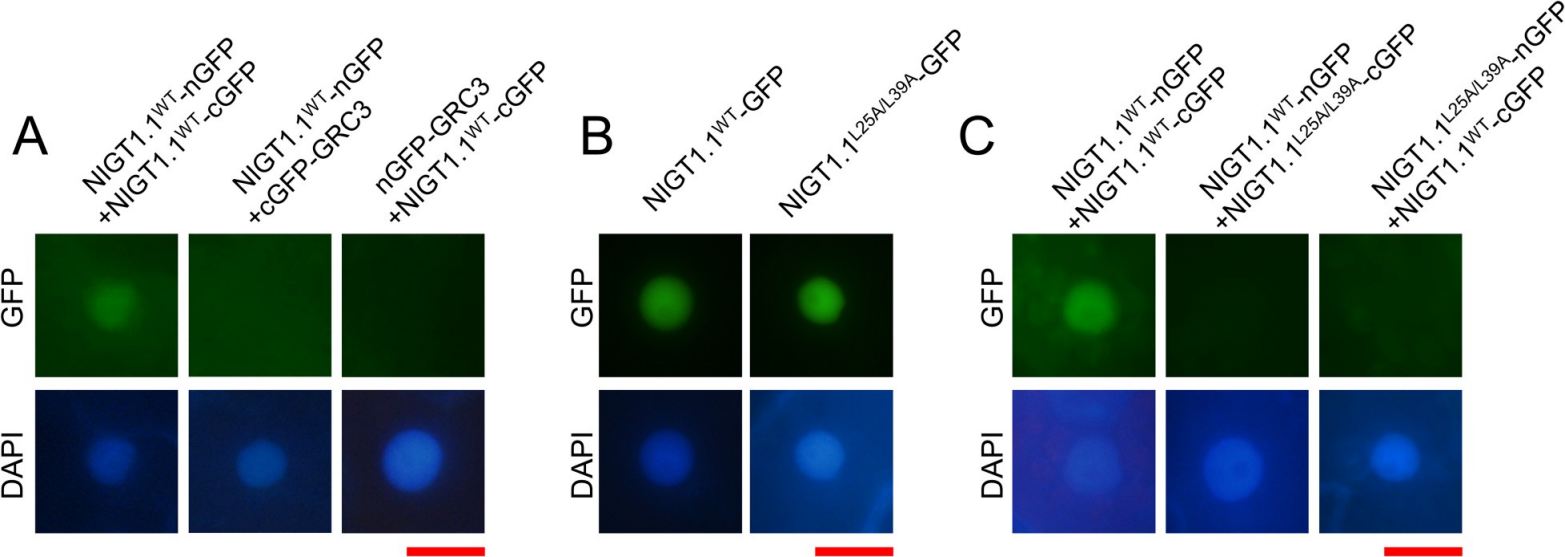
NIGT1–NIGT1 interaction *in planta*. **(A)** Bimolecular fluorescence complementation (BiFC) assays to test NIGT1-NIGT1 interaction in *N*. *benthamiana* leaves by agroinfiltration. The N-terminal or C-terminal region of GFP (nGFP and cGFP, respectively) was fused to NIGT1.1^WT^ or a nuclear protein GRC3 (negative control). GFP fluorescence was observed 3 d post-agroinfiltration. **(B)** Subcellular localization of NIGT1.1^WT^ and NIGT1.1^L25A/L39A^ in *N*. *benthamiana* leaves. NIGT1.1^WT^-GFP and NIGT1.1^L25A/L39A^-GFP translational fusion proteins were expressed in *N*. *benthamiana* leaves by agroinfiltration, and GFP fluorescence was observed after 2 d. **(C)** Effects of amino acid substitutions in the CCD on NIGT1–NIGT1 interaction *in planta*. Translational fusions of NIGT1.1^WT^ or NIGT1.1^L25A/L39A^ with nGFP or cGFP were co-expressed in *N*. *benthamiana* leaves by agroinfiltration, and GFP fluorescence was examined after 3 d. In **(A–C)**, nuclei were stained with DAPI prior to observation. Scale bars: 20 μm.

To verify the results of Y2H assays *in planta*, we performed bimolecular fluorescence complementation (BiFC) assays using NIGT1.1 fused to the N-terminal or C-terminal half of the green fluorescent protein (NIGT1.1-nGFP and NIGT1.1-cGFP, respectively). We focused on NIGT1.1 in this experiment because *NIGT1*.*1* is expressed both in shoots and roots [26], and complements both shoot- and root-related phenotypes of the *nigt1*.*1*/*nigt1*.*2*/*nigt1*.*3*/*nigt1*.*4* quadruple mutant (hereafter referred to as the *nigtQ* mutant) [30]. Co-expression of NIGT1.1^WT^-nGFP with NIGT1.1^WT^-cGFP in *Nicotiana benthamiana* leaves resulted in GFP fluorescence in the nuclei (Fig 2A). However, fluorescence was not observed when NIGT1.1^WT^-nGFP or NIGT1.1^WT^-cGFP was co-expressed with cGFP or nGFP fusion of an unrelated nuclear-localizing protein GRC3 [40] (Fig 2A). Furthermore, although nuclear localization of NIGT1.1^WT^ and NIGT1.1^L25A/L39A^ was confirmed by fusing them to full-length GFP (Fig 2B), NIGT1.1^L25A/L39A^ failed to interact with NIGT1.1^WT^ in leaf cells (Fig 2C). These results demonstrate the interaction among NIGT1 family proteins via the N-terminal CCD in plant cell nuclei, and confirm that the two Leu residues in the CCD are essential for this interaction.

### NIGT1.1 forms a dimer *via* the CCD in solution

**Fig 3:**
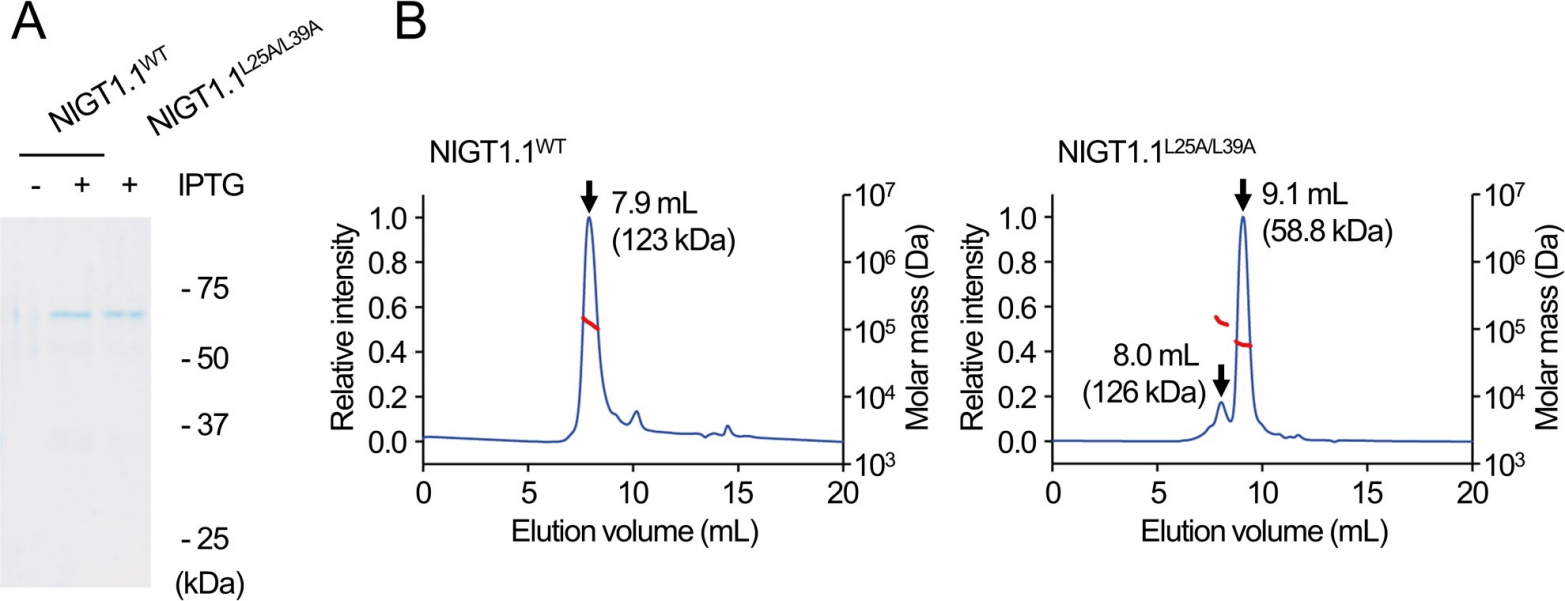
Effects of amino acid substitutions within the CCD of NIGT1.1 on the complex formation. **(A)** Analysis of purified recombinant NIGT1.1^WT^ and NIGT1.1^L25A/L39A^ proteins with a SDS-containing polyacrylamide gel. Protein extracts were prepared from *E*. *coli* cells treated with (+) or without (-) IPTG. Proteins were stained with Coomassie Brilliant Blue. Numbers indicate the molecular weight of standard proteins run on the same gel. **(B)** Size exclusion chromatography coupled to multi angle light scattering (SEC-MALS) analysis using purified recombinant NIGT1.1^WT^ and NIGT1.1^L25A/L39A^ proteins. The chromatogram displays the relative intensity of absorbance at 280 nm (blue, left axis) together with the molar mass of the peak calculated by MALS (red, right axis). The elution volume and the average molar mass of the protein for each peak are indicated.

We hypothesized that the loss of protein–protein interaction ability of NIGT1 family proteins would interfere with the NIGT1–NIGT1 complex formation, thus affecting their dual mode DNA recognition ability. To test this hypothesis, we produced histidine (His)- and thioredoxin (Trx)-tagged NIGT1.1^WT^ and NIGT1.1^L25A/L39A^ proteins in *Escherichia coli* using pET32 plasmid, and purified these recombinant proteins (Fig 3A). It is important to note that the bacterial Trx exists as a monomer [41]. Size exclusion chromatography coupled to multi-angle scattering (SEC-MALS) analysis was performed to determine the absolute molecular masses of recombinant NIGT1.1^WT^ and NIGT1.1^L25A/L39A^ proteins in solution (Fig 3B). In this analysis, NIGT1.1^WT^ protein produced a single peak corresponding to a molecular mass of 123 kDa, which is approximately twice the expected molecular mass of recombinant NIGT1.1 protein (57.4 kDa). On the other hand, NIGT1.1^L25A/L39A^ protein was mostly eluted at a peak corresponding to a molecular mass of 58.8 kDa. These results revealed that NIGT1.1^WT^ exclusively exists as a dimer, while most of NIGT1.1^L25A/L39A^ exists as a monomer.

### Disruption of the CCD affects dual mode DNA recognition by NIGT1.1

**Fig 4:**
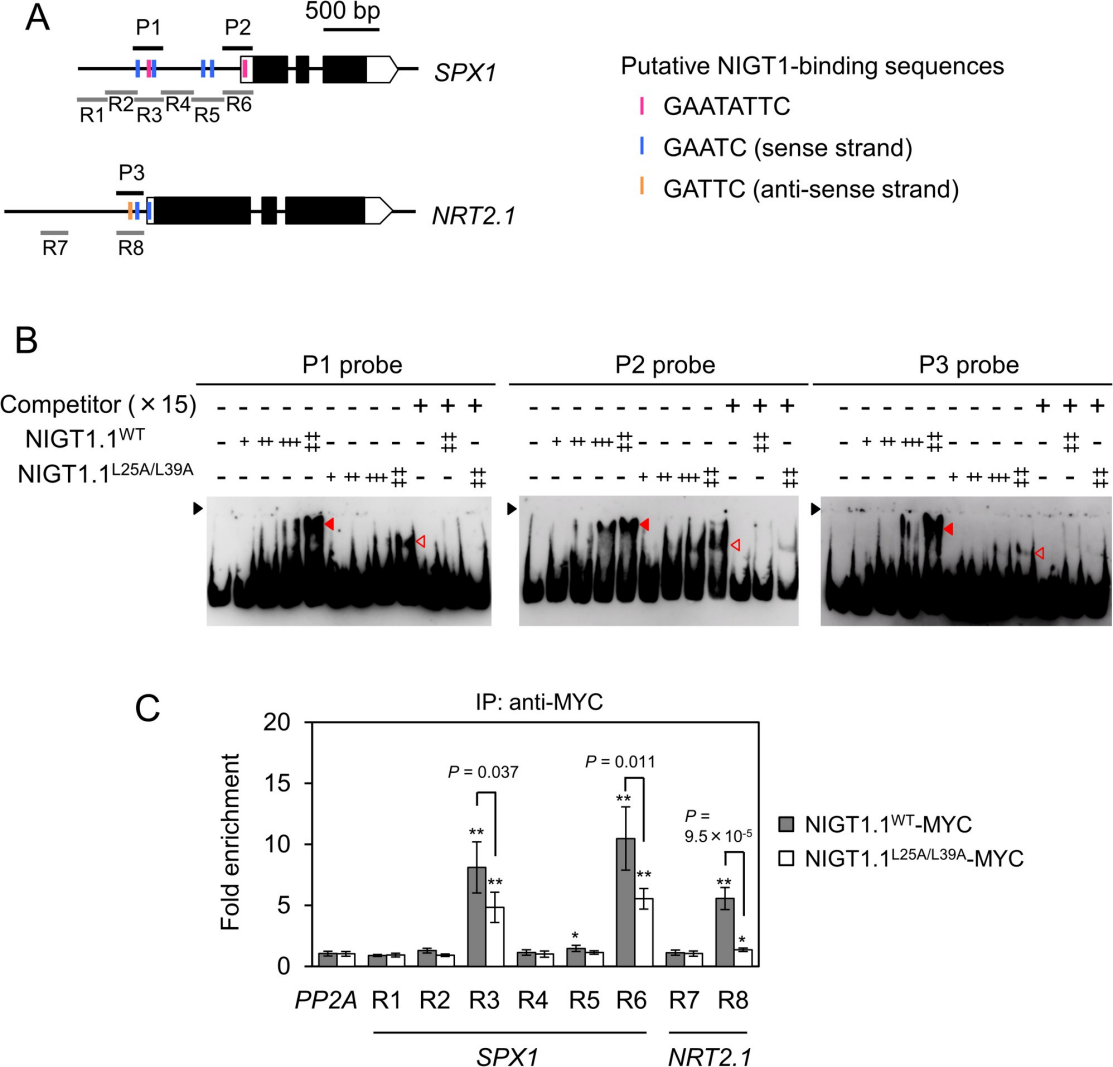
Effects of amino acid substitutions within the CCD on the DNA-binding ability of NIGT1.1. **(A)** Structure of *SPX1* and *NRT2*.*1* genes. Red vertical line in the promoter regions indicate the position of palindromic (GAATATTC) motif, and blue and purple vertical lines indicate non-palindromic (GAATC) motifs present in the sense and anti-sense strands, respectively. Black squares indicate the coding sequences, and white squares and pentagons indicate 5' untranslated region (5'UTR) and 3'UTR, respectively. Black horizontal lines indicate the positions of probes (P1–P3) used to perform electrophoretic mobility shift assays (EMSAs) in **(B)**. Gray horizontal lines indicate the regions (R1–R8) used for ChIP assay in **(C)**. **(B)** EMSA. In each panel, 0, 7.5, 15, 30, or 60 ng of the purified protein was mixed with a DNA probe indicated in **(A)** in the presence or absence of the indicated molar excess of competitor DNA. NIGT1.1^WT^-DNA and NIGT1.1^L25A/L39A^-DNA complexes are indicated by closed and open red arrows, respectively. Black arrow indicates the position of the origin of electrophoresis. **(C)** Chromatin immunoprecipitation (ChIP) assay. Binding of NIGT1.1^WT^ and NIGT1.1^L25A/L39A^ to specific regions in *SPX1* and *NRT2*.*1* gene promoters was assessed using 12-d-old seedlings of *nigtQ*/NIGT1.1^WT^ (#8) and *nigtQ*/NIGT1.1^L25A/L39A^ (#11). Data represent mean ± SD (*n* = 4). *PP2A* served as a negative control. Two-tailed Student’s *t*-test was conducted to determine significant differences in the fold enrichment between *PP2A* and each DNA region, and the significance is indicated as follows; *, *P* < 0.05; **, *P* < 0.001. For region R3, R6, and R8, fold enrichment was further compared between NIGT1.1^WT^-MYC and NIGT1.1^L25A/L39A^-MYC by two-tailed Student’s *t*-test, and *P* values are indicated.

To test whether the inability of NIGT1 proteins to form dimers affects their dual mode DNA recognition and binding ability, we performed electrophoretic mobility shift assays (EMSAs) using recombinant NIGT1.1^WT^ and NIGT1.1^L25A/L39A^ proteins. Previous studies showed that NIGT1 family proteins bind to specific sequences in *SPX1* and *NRT2*.*1* gene promoters, and also revealed the physiological significance of this binding [24,30]. Based on the information from these studies, we used three DNA probes (P1–P3) corresponding to the authentic NIGT1 binding regions that were experimentally identified in *SPX1* and *NRT2*.*1* gene promoters: P1 probe, amplified from the *SPX1* promoter, contained two copies of the non-palindromic motif (GAATC or its reverse complement, GATTC) and one copy of the palindromic motif (GAATATTC); P2 probe, also amplified from the *SPX1* promoter, contained only one copy of the palindromic motif; and P3 probe, amplified from the *NRT2*.*1* promoter, contained two copies of the non-palindromic motif (Fig 4A). Consistent with previous results, the binding of NIGT1.1^WT^ to these probes was inhibited by the presence of excessive amounts of non-labeled probes as competitors (Fig 4B). Protein–DNA complexes comprising NIGT1.1^L25A/L39A^ migrated faster in the gel compared with those comprising the NIGT1.1^WT^ protein, as expected, because of their lower molecular weight. Furthermore, the amount of NIGT1.1^L25A/L39A^–DNA complexes was less than that of the NIGT1.1^WT^–DNA complex, suggesting the compromised affinity of the NIGT1.1^L25A/L39A^ toward any of the three DNA probes. These results suggest that dimerization is required for the dual mode, high affinity DNA-binding ability of NIGT1 family proteins.

To compare DNA binding of NIGT1.1^WT^ and NIGT1.1^L25A/L39A^ *in vivo*, we performed chromatin immunoprecipitation (ChIP) assays using *nigtQ* plants that constitutively expressed MYC epitope-tagged NIGT1.1^WT^ or NIGT1.1^L25A/L39A^ (*nigtQ*/NIGT1.1^WT^ #8 and *nigtQ*/NIGT1.1^L25A/L39A^ #11). Although both of *NIGT1*.*1* transcript and NIGT1.1 protein levels were comparable in these transgenic plants (S3 Fig), the result of ChIP assays suggested the differences in *in vivo* binding of NIGT1.1^WT^ and NIGT1.1^L25A/L39A^ to NIGT1 binding sites in regions R3, R6 and R8 of the *SPX1* or *NRT2*.*1* promoters (Fig 4A and 4C).

### Unique mode of DNA recognition by NIGT1.1

**Fig 5:**
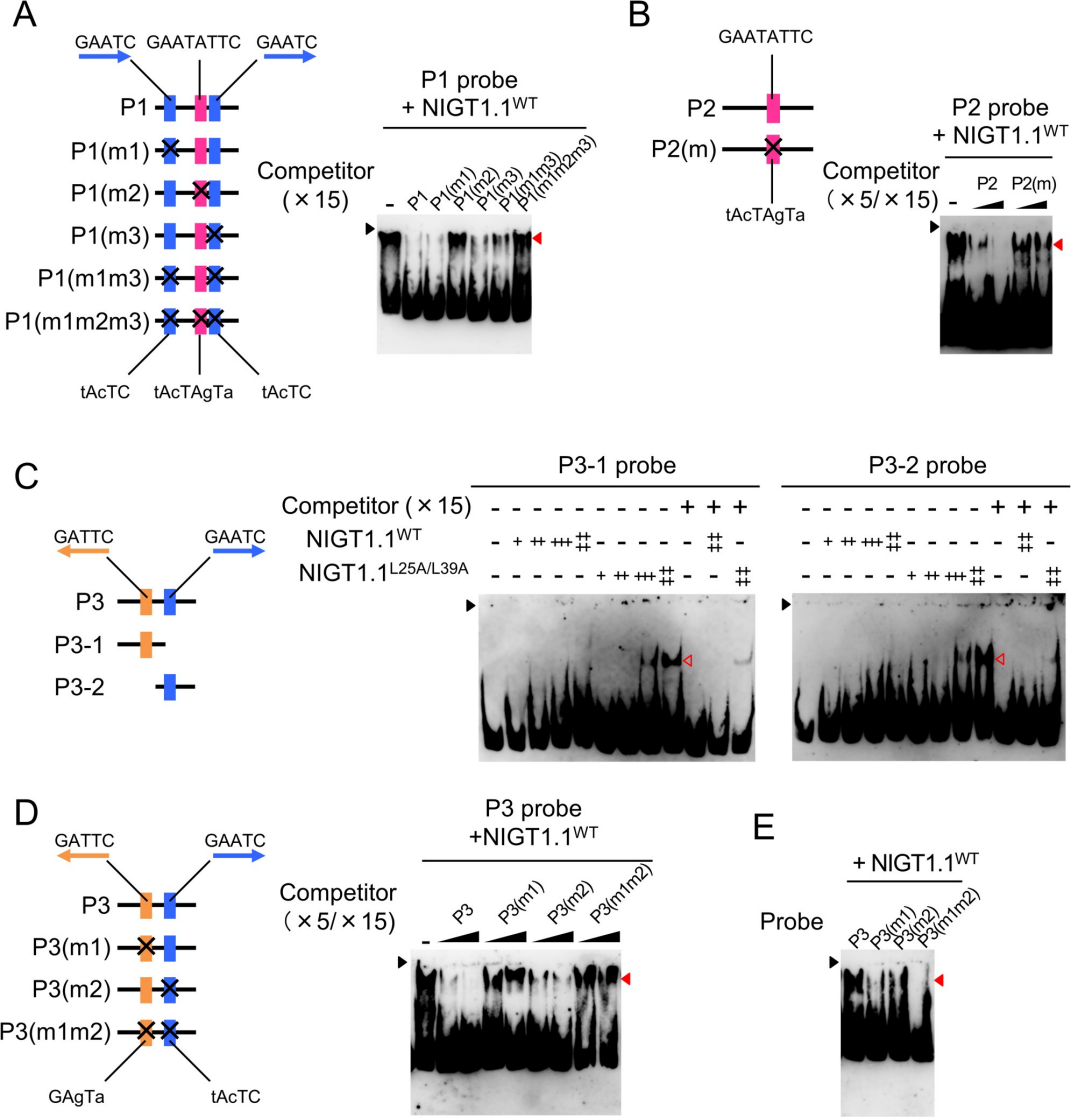
Dual mode binding of NIGT1.1 to the sequences in *SPX1* and *NRT2*.*1* gene promoters. **(A, B)** EMSAs using non-labeled competitor DNA carrying mutations at each of the putative NIGT1-binding sites in P1 **(A)** or P2 **(B)** probe (see Fig 4A). Each reaction mixture contained 60 ng of the purified protein and labeled P1 or P2 probe. **(C)** EMSAs using DNA probes obtained by the dissection of probe P3. Each probe carried only a single copy of the non-palindromic NIGT1-binding sequence. In each panel, 0, 7.5, 15, 30, and 60 ng of purified NIGT1.1^WT^ or NIGT1.1^L25A/L39A^ protein was incubated in the presence or absence of the indicated molar excess of competitor DNA. **(D)** EMSA using the P3 probe, non-labeled mutant P3 probes (competitor DNAs), and 60 ng of purified NIGT1.1^WT^. **(E)** EMSAs using mutant P3 probes and 60 ng of purified protein. In **(A–E)**, closed and open red arrows indicate NIGT1.1^WT^-DNA and NIGT1.1^L25A/L39A^-DNA complexes, respectively. Black arrow indicates the position of the origin of electrophoresis. Schematic representations of the structure and positions of mutations are shown in each panel.

NIGT1.1^WT^, which exclusively existed as a dimer, bound to the P3 probe containing only non-palindromic motifs more effectively, compared with NIGT1.1^L25A/L39A^ that existed as a monomer (Fig 4). To clarify the reason for this unexpected observation, we performed competition assays using non-labeled competitor DNAs harboring mutations at different NIGT1-binding sites within the P1 sequence that contained both non-palindromic and palindromic motifs (Fig 5A). The competitor DNA harboring mutations in the palindromic motif, designated as non-labeled P1(m2), did not inhibit the interaction between labeled P1 probe and NIGT1.1^WT^, while competitor DNAs with mutations in the non-palindromic motif, designated as non-labeled P1(m1), (m3), and (m1m3), competed potently with the labeled P1 probe (Fig 5A). Hence, NIGT1.1^WT^ recognized only the palindromic motif in the P1 probe. Similar results were obtained using non-labeled mutant variants of the P2 probe (Fig 5B).

The NIGT1.1^WT^ protein effectively bound to non-palindromic motifs in the P3 probe but not to non-palindromic motifs in the P1 probe. To resolve this conflicting result, binding of NIGT1.1^WT^ to a single non-palindromic motif was examined by EMSA using DNA sequences dissected from the P3 probe (P3-1 and P3-2), which contained only a single copy of the non-palindromic motif. The results showed that NIGT1.1^WT^ was unable to bind to P3-1 and P3-2 probes containing only one non-palindromic NIGT1-binding motif, whereas NIGT1.1^L25A/L39A^ could bind to these probes (Fig 5C). The P1 probe contained two tandem repeats of the non-palindromic GAATC motif, whereas the P3 probe contained two copies of the non-palindromic GAATC motif in the reverse orientation. Thus, it was likely that each NIGT1.1^WT^ subunit of the NIGT1.1^WT^ dimer recognized one GAATC motif in the reverse orientation. To corroborate this hypothesis, competition assays were performed using non-labeled mutant P3 probe in which either one or both non-palindromic NIGT1-binding sequences were mutated (Fig 5D). Non-labeled competitor DNAs harboring mutations at either of these sites [P3(m1) and (m2)] or both sites [P3(m1m2)] competed less effectively with the labeled P3 probe than the wild-type competitor DNA. Consistent results were obtained in EMSAs using labeled P3(m1), (m2), and (m1m2) probes (Fig 5E). Hence, we conclude that a single copy of the GAATC motif is not sufficient for the NIGT1^WT^–DNA interaction; however, the NIGT1.1 dimer can bind to two copies of the GAATC motif in the reverse orientation, even if they are separated by a spacer sequence (a 38 bp sequence in probe P3), and then form a stable protein–DNA complex through the interaction of each subunit of the dimer with the GAATC motif. Thus, dual mode DNA recognition by NIGT1 proteins is not conferred by the recognition of GAATC and GAATATTC sequences but by the recognition of GAATATTC and GATTC-N_38_-GAATC sequences, revealing the uniqueness of dual mode DNA recognition by NIGT1 family proteins (described in further detail in the Discussion). On the other hand, NIGT1.1^L25A/L39A^ bound to a single copy of the GAATC motif with low affinity as a monomer.

### Different modulations of the expression of nutrient response-associated genes by NIGT1.1^WT^ and NIGT1.1^L25A/L39A^ *in vivo*

**Fig 6:**
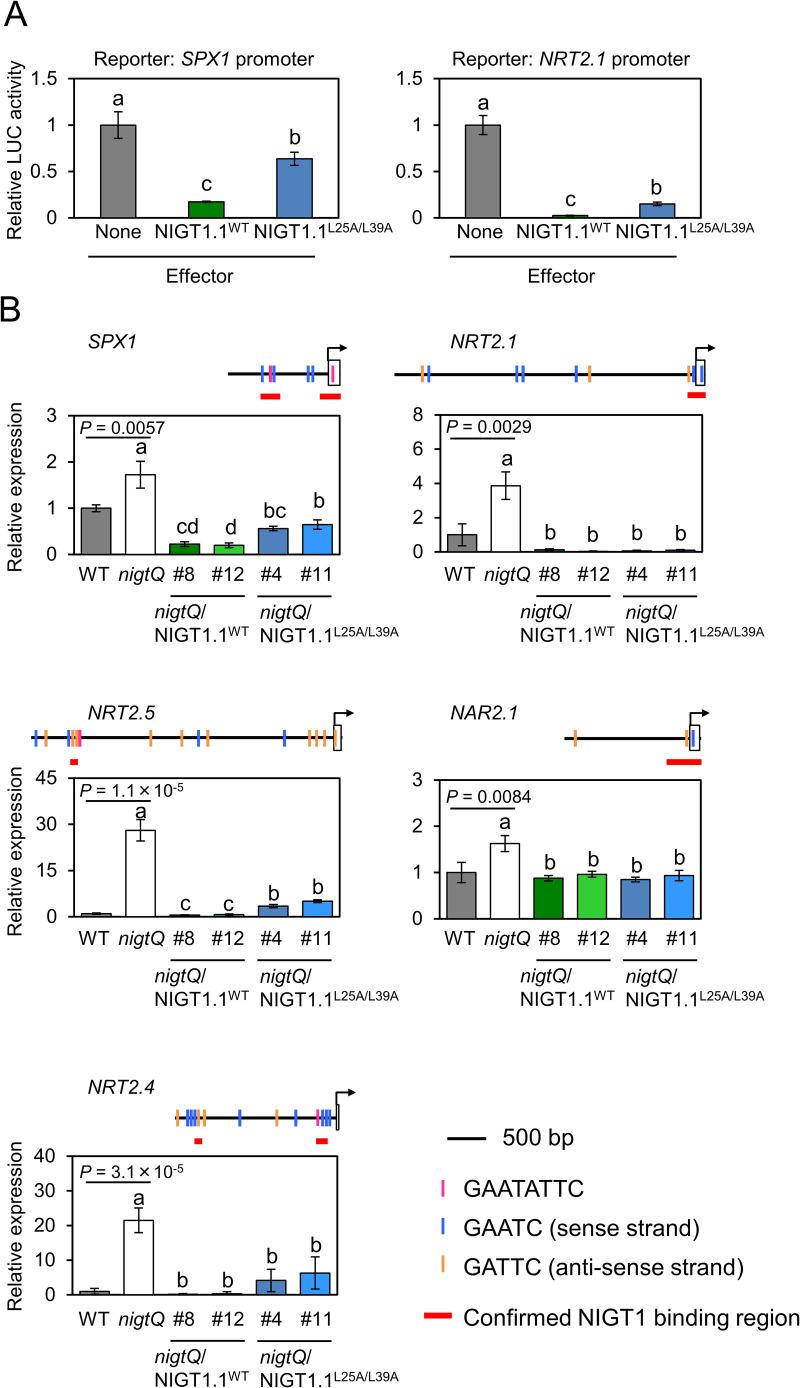
Effects of mutations within the NIGT1.1 CCD on target gene transcription *in vivo*. **(A)** Co-transfection assay. An effector plasmid used to express NIGT1.1^WT^ or NIGT1.1^L25A/L39A^ was co-transfected into Arabidopsis protoplasts together with a reporter plasmid harboring the *LUC* gene under the control of the *SPX1* or *NRT2*.*1* promoter and an internal control plasmid containing the *GUS* gene. LUC activity was normalized relative to GUS activity. Data represent mean ± standard deviation (SD; *n* = 4–5) of relative LUC activity. The value obtained with an empty effector plasmid (none) was set to 1 in each panel. Significant differences among values obtained with different effector plasmids were determined using one-way ANOVA, followed by Tukey’s HSD test, and are indicated by different lowercase letters. **(B)** Expression of NIGT1 target genes in roots of transgenic *nigtQ* plants expressing NIGT1.1^WT^ or NIGT1.1^L25A/L39A^ (*nigtQ*/NIGT1.1^WT^ and *nigtQ*/NIGT1.1^L25A/L39A^). Expression levels were analyzed in roots of 9-d-old plants grown on agar plates containing Pi-replete medium. Data represent mean ± SD (*n* = 4). Values in each sample were normalized relative to those in wild-type (WT) plants. Structure of the promoter region of each gene (up to the immediately adjacent gene, or 4 kb sequence upstream from the translational start codon) is indicated, with the right end corresponding to the start codon. White squares and arrows indicate 5’UTRs and the positions of transcription start sites, respectively. NIGT1 binding motifs are indicated. Significant differences between WT and *nigtQ* plants under each condition were determined using two-tailed Student’s *t*-test. *P* values are indicated. Significant differences among the *nigtQ*, *nigtQ*/NIGT1.1^WT^, and *nigtQ*/NIGT1.1^L25A/L39A^ lines were determined using one-way ANOVA, followed by Tukey’s HSD test, and are indicated by different lowercase letters.

Amino acid substitutions within the CCD of NIGT1.1 did not affect its nuclear localization (Fig 2B). Furthermore, intact and truncated NIGT1.1 proteins fused to the GAL4 DNA-binding domain similarly repressed the GAL4-binding site-containing synthetic promoter (S4 Fig), indicating that NIGT1.1 represses transcription independently of the N-terminal CCD as long as NIGT1.1 can bind to target DNA sequences. Thus, NIGT1.1^L25A/L39A^ was assumed to possess no or reduced activity for repression of target gene promoters, due to its reduced affinity to target DNA sequences. Co-transfection assays using Arabidopsis protoplasts revealed that the NIGT1.1^L25A/L39A^ protein still repressed both *SPX1* and *NRT2*.*1* promoters; however, as hypothesized, the repression by the mutant NIGT1.1^L25A/L39A^ was weaker than that by NIGT1.1^WT^ (Fig 6A), consistent with the lower ability of NIGT1.1^L25A/L39A^ to bind to *SPX1* and *NRT2*.*1* promoters (Fig 4B). We note that the weaker repressor activity of NIGT1.1^L25A/L39A^ was not likely caused by its lower stability, since its half-life was not shorter than that of NIGT1.1^WT^ in plant cells (S5 Fig).

To further reveal the physiological consequences of the defect in NIGT1 dimerization *in planta*, gene expression analysis was performed using RNA extracted from the roots of wild-type, *nigtQ*, *nigtQ*/NIGT1.1^WT^ and *nigtQ*/NIGT1.1^L25A/L39A^ plants. Levels of *NIGT1*.*1* transcripts and NIGT1.1 proteins were similar in two independent transgenic lines in each of *nigtQ*/NIGT1.1^WT^ and *nigtQ*/NIGT1.1^L25A/L39A^ genotype (S3 Fig). Plants were grown under the Pi-replete condition, because the effect of *nigtQ* mutation on *SPX1* expression was more evident under this condition than under the Pi-deplete condition [30]. NIGT1.1^L25A/L39A^ suppressed *SPX1* expression but to a lesser extent than NIGT1.1^WT^; by contrast, NIGT1.1^L25A/L39A^ and NIGT1.1^WT^ repressed the expression of *NRT2*.*1* to similar levels (Fig 6B). This is consistent with the observation that L25A and L39A amino acid substitutions in the CCD of NIGT1.1 exerted negative effects on NIGT1.1-mediated repression of *SPX1* and *NRT2*.*1* promoters to different extents; NIGT1.1^L25A/L39A^ retained only 44% of the repression activity of NIGT1.1^WT^ on the *SPX1* promoter but 87% of the repression activity on the *NRT2*.*1* promoter (Fig 6A). Furthermore, since NIGT1 binding regions in the promoters of three nitrate transporter genes including *NRT2*.*4*, *NRT2*.*5*, and *NAR2*.*1* had been identified by ChIP analysis [24,26], expression levels of these genes in *nigtQ*/NIGT1.1^WT^ and *nigtQ*/NIGT1.1^L25A/L39A^ plants were also examined. The result further clarified that diminishing the ability for dimerization exerts different effects on the expression levels of distinct target genes *in planta* (Fig 6B). Interestingly, the result suggested that disruption of the CCD had no substantial effect on the NIGT1.1-meditated repression of the *NRT2*.*1* and *NAR2*.*1* promoters that lacked the palindromic GAAGATTC motif, while NIGT1.1^WT^ and NIGT1.1^L25A/L39A^ differentially repressed transcription from the *SPX1*, *NRT2*.*4*, and *NRT2*.*5* promoters that contained at least one functional GAAGATTC motif.

### Abolishing the NIGT1 dimerization compromises nutrient responses *in planta*

**Fig 7:**
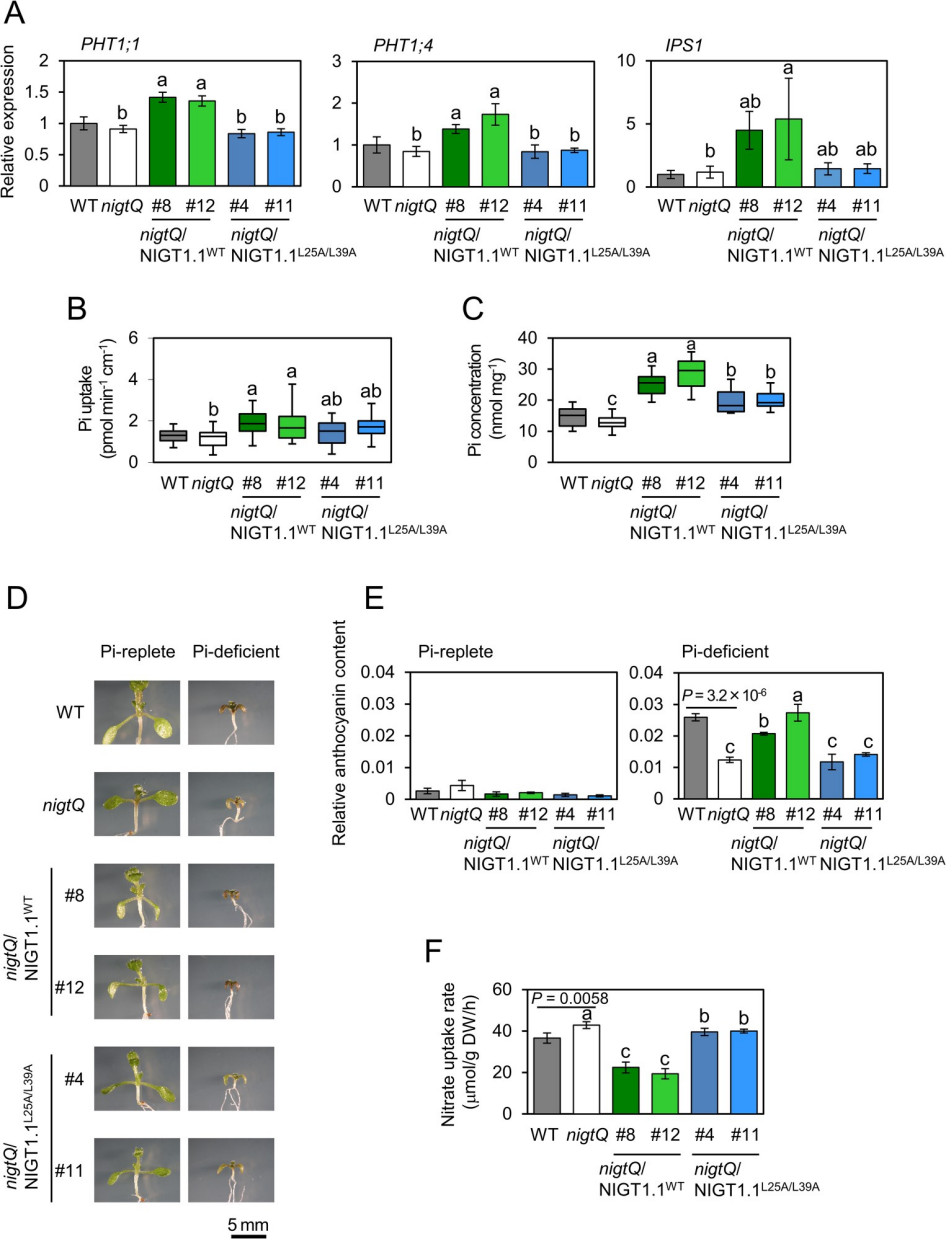
Effects of impaired NIGT1–NIGT1 interaction on the nutrient response-related phenotype. **(A)** Expression analysis of *PHT1;1* and *PHT1;4* in roots of 9-d-old WT, *nigtQ*, *nigtQ*/NIGT1.1^WT^, and *nigtQ*/NIGT1.1^L25A/L39A^ seedlings grown on agar plates containing Pi-replete medium. Data represent mean ± SD (*n* = 4). **(B)** Analysis of Pi uptake in WT, *nigtQ*, *nigtQ*/NIGT1.1^WT^, and *nigtQ*/NIGT1.1^L25A/L39A^ seedlings. The value of ^33^P-radiolabeled Pi taken up by each 9-d-old seedling was divided by the total root length (i.e., sum of lengths of the primary root and all lateral roots). Seedlings were grown on agar plates containing Pi-replete medium. **(C)** Free Pi concentrations in the shoot of 9-d-old WT, *nigtQ*, *nigtQ*/NIGT1.1^WT^, and *nigtQ*/NIGT1.1^L25A/L39A^ seedlings grown on agar plates containing Pi-replete medium. **(D)** Foliar anthocyanin accumulation in 9-d-old WT, *nigtQ*, *nigtQ*/NIGT1.1^WT^, and *nigtQ*/NIGT1.1^L25A/L39A^ seedlings grown in Pi-replete or Pi-deficient liquid medium. Scale bar = 5 mm. **(E)** Anthocyanin contents of WT, *nigtQ*, *nigtQ*/NIGT1.1^WT^, and *nigtQ*/NIGT1.1^L25A/L39A^ plants grown under the same condition as **(D)**. Data represent mean ± SD (*n* = 4). **(F)** Nitrate uptake in 10-d-old WT, *nigtQ*, *nigtQ*/NIGT1.1^WT^, and *nigtQ*/NIGT1.1^L25A/L39A^ seedlings grown on agar plates containing Pi-replete medium. Data represent mean ± SD (*n* = 4). In **(A–C, E and F)**, *nigtQ*, *nigtQ*/NIGT1.1^WT^, and *nigtQ*/NIGT1.1^L25A/L39A^ lines were statistically compared using one-way ANOVA, and different lowercase letters indicate significant differences indicated by Tukey’s HSD test. Two-tailed Student’s *t*-test was conducted to determine significant differences between WT and *nigtQ* plants under each condition, and the *P* values are indicated. In box plots **(B, C)**, the middle horizontal bar indicates the median, and the upper and lower ends of the box indicate the upper and lower quantiles, respectively. *n* = 18–22 in **(B)** and 9–10 in **(C)**.

We previously showed that NIGT1 family proteins modulate Pi uptake and PSR via the repression of *SPX1* [30]. Therefore, physiological significance of dimerization of NIGT1 family proteins was first examined through the analysis of Pi uptake and PSR-related phenotype of *nigtQ*/NIGT1.1^L25A/L39A^ transgenic lines. Since genes encoding Pi transporters that play a major role in Pi uptake from roots, *PHT1;1* and *PHT1;4* [42], are negatively regulated by SPX proteins during PSR [17], we analyzed the expression levels of these genes. The results showed that *PHT1;1* and *PHT1;4* transcript levels in *nigtQ* plants were lower than in *nigtQ*/NIGT1.1^WT^ lines but comparable to those in *nigtQ*/NIGT1.1^L25A/L39A^ lines (Fig 7A). Furthermore, similar to *PHT1;1* and *PHT1;4* transcript levels, expression levels of *IPS1*, a marker gene for PSR [5], were modified in *nigtQ* plants, *nigtQ*/NIGT1.1^WT^, and *nigtQ*/NIGT1.1^L25A/L39A^ plants (Fig 7A). These modifications were consistent with the reduced suppression of *SPX1* expression in *nigtQ*/NIGT1.1^L25A/L39A^ lines (Fig 6B). Consistently, Pi uptake activity (Fig 7B) and shoot Pi concentration (Fig 7C) in *nigtQ*/NIGT1.1^L25A/L39A^ lines were intermediate between *nigtQ* and *nigtQ*/NIGT1.1^WT^ lines. Next, the PSR of *nigtQ*/NIGT1.1^L25A/L39A^ lines was evaluated by Pi starvation-induced accumulation of anthocyanins (Fig 7D and 7E). Under Pi-deficient conditions, L25A and L39A amino acid mutations within the CCD of NIGT1.1 clearly attenuated anthocyanin accumulation. While *nigtQ*/NIGT1.1^WT^ lines showed a significant increase in the anthocyanin content compared with *nigtQ* plants, no substantial increase was observed in *nigtQ*/NIGT1.1^L25A/L39A^ lines (Fig 7D and 7E).

Differences in nitrate uptake and metabolism-related physiological traits of *nigtQ*/NIGT1.1^WT^ and *nigtQ*/NIGT1.1^L25A/L39A^ plants were also investigated. The *nigtQ*/NIGT1.1^WT^ plants showed greatly reduced nitrate uptake, compared with *nigtQ* plants, while the *nigtQ*/NIGT1.1^L25A/L39A^ plants showed only minorly reduced nitrate uptake (Fig 7F), consistent with differential repression of nitrate transporter genes in *nigtQ*/NIGT1.1^WT^ and *nigtQ*/NIGT1.1^L25A/L39A^ plants (Fig 6). Furthermore, a clear contrast in shoot growth was observed between the *nigtQ*/NIGT1.1^WT^ and *nigtQ*/NIGT1.1^L25A/L39A^ plants when grown with nitrate as the sole N source, while difference in shoot growth was not obvious between ammonium-grown *nigtQ*/NIGT1.1^WT^ and *nigtQ*/NIGT1.1^L25A/L39A^ plants (S6 Fig). These results suggest the significance of dimerization of NIGT1 family proteins in controlling nitrate use.

We also found that when grown on the soil, retardation of shoot growth and accumulation of Pi were evident in *nigtQ*/NIGT1.1^WT^ plants but not in *nigtQ*/NIGT1.1^L25A/L39A^ plants (S7 Fig). Thus, the defect in dimerization of NIGT1 proteins likely causes altered nutrient responses under various growth conditions.

## Discussion

In the current study, we revealed that the domain containing a coiled-coil motif in the N-terminal region of NIGT1 family proteins mediates homologous and heterologous NIGT1–NIGT1 interactions, and plays a key role in dual mode recognition of *cis*-elements in target gene promoters by NIGT1 family proteins. Since NIGT1 family proteins are key regulators of nitrate response and PSR [24,26–30], and the physiological significance of dimerization of NIGT1 family proteins was confirmed in the current study, the findings of this study are important for a deeper understanding of the regulation of nutrient responses in plants. Moreover, these findings also provide novel insights into how plant TFs evolutionarily gained the ability to recognize diverse DNA sequences.

### Amino acid sequence of the CCD is highly conserved among NIGT1 family proteins

The CCD enables dual mode DNA recognition of *cis*-elements by NIGT1 family proteins and ensures proper regulation of the responses to nitrate and Pi. A search for the CCD sequence in the public database containing diverse genetic resources, namely, the Arabidopsis 1001 Genomes database (https://1001genomes.org/tools.html; last accessed in Jan 2020), which contains CCD sequences from 1,135 naturally occurring accessions [43], indicated that the core 22-aa sequence of the CCD is very highly conserved. Sequence conservation of the CCD is much higher than that of flanking regions and even higher than that of the GARP DNA-binding domain (S8 Fig). Furthermore, although amino acid substitutions were detected in these sequences at glutamate or lysine residues within the CCD, no substitutions of branched-chain amino acid residues were detected in the sequence that constitutes the hydrophobic surface of the coiled-coil motif. Similarly, analysis using the rice SNP-Seek database (https://snp-seek.irri.org/; last accessed in Jan 2020) to identify sequence variants in 3,024 rice accessions [44] revealed no amino acid substitutions within the CCD of OsNIGT1 (S8 Fig). These data suggest that the CCD of NIGT1 family proteins has been highly conserved during evolution, perhaps because CCD-mediated dimerization of NIGT1 family proteins is indispensable for some aspects of NIGT1-mediated transcriptional regulation and proper nutrient responses.

### The N-terminal CCD confers a unique mode of DNA recognition to NIGT1 family proteins

**Fig 8:**
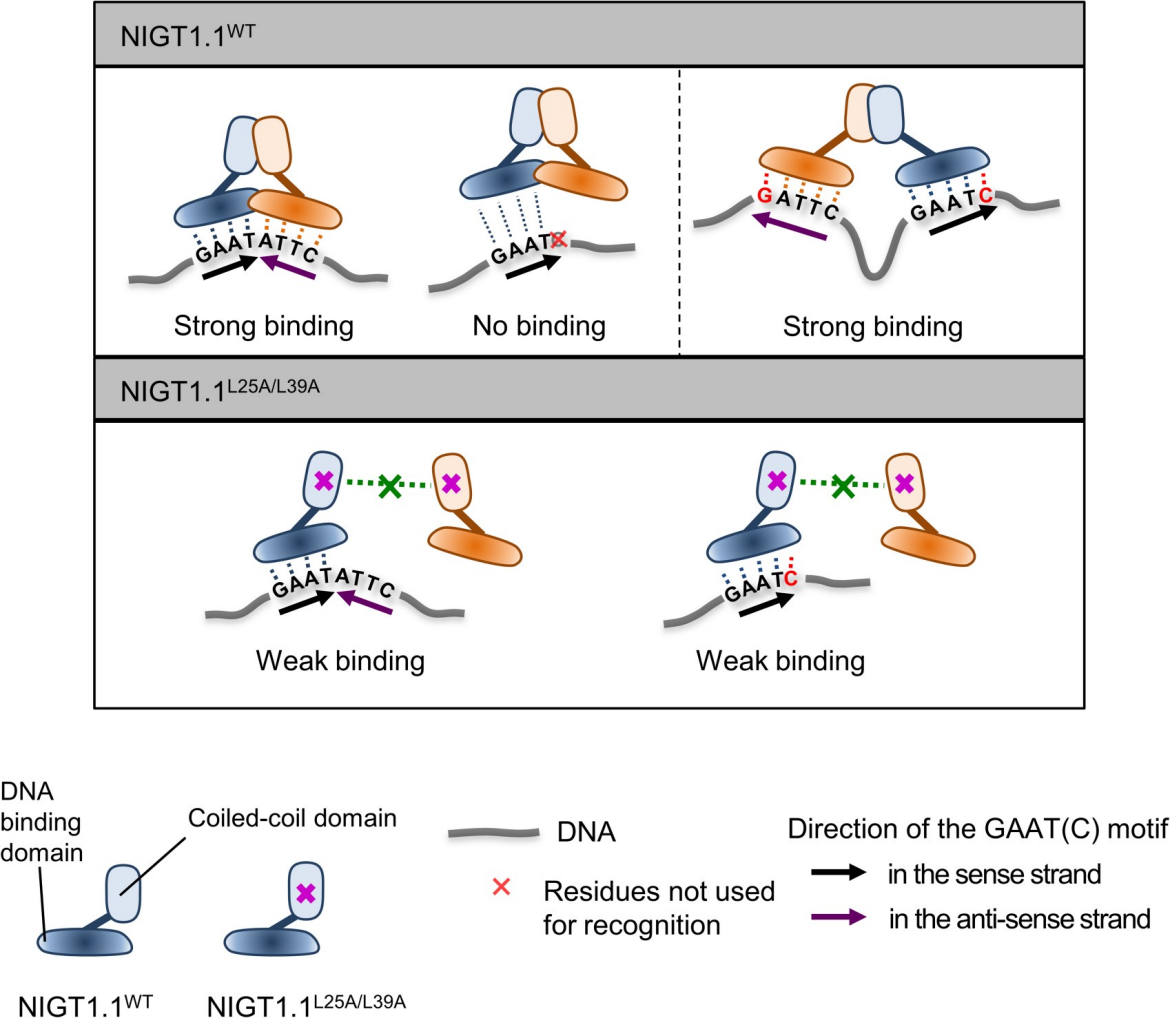
Model displaying the possible modes of NIGT1–DNA interaction. NIGT1 binding to a single palindromic and two separate non-palindromic sites. Dimeric NIGT1 binds to the palindromic GAATATCC sequence with high affinity but not to a single copy of the GAATC sequences, while monomeric mutant NIGT1 binds to a single copy of the GAATC sequences with low affinity. Dimeric NIGT1 binds to the GATTC-N_x_-GAATC sequences with high affinity through recognition of a single GAATC motif by each subunit. Each subunit of the NIGT1 dimer is indicated in blue or brown.

Protein–protein interactions play important roles in the transduction of environmental signals and are important for proper function of enzymes and transporters. In some TF families, protein dimerization and/or oligomerization play crucial roles in the recognition of target DNA sequences and/or proper regulation of target genes [45–48]. For instance, in durum wheat (*Triticum turgidum*), a LATERAL ORGAN BOUNDARIES DOMAIN (LBD) family TF, TtRa2LD, forms a homodimer to increase the affinity for target *cis*-elements [47]. Similarly, dimerization of Arabidopsis AUXIN RESPONSE FACTORS (ARFs) enhances their affinity for target DNA sequences [46]. Furthermore, it was proposed that dimerization of eukaryotic TFs emerged during evolution to allow the recognition of a larger number of nucleotides [49]. In the NIGT1 family, dimerization is necessary for binding to two types of palindromic DNA sequences (i.e. GAATATTC and GATTC-N_38_-GAATC) with high affinity (Fig 8). In this study, we showed that NIGT1.1^WT^ could not bind to a single GAATC motif, whereas NIGT1.1^L25A/L39A^ could bind to this sequence (Fig 8) probably because the NIGT1.1^L25A/L39A^ protein bares additional amino acid residues required for DNA recognition on the surface, which were buried within the interaction surface of the NIGT1.1^WT^ dimer, as hypothesized previously [32] (Fig 8); however, the affinity and specificity of NIGT1.1^L25A/L39A^ for a single GAATC motif did not appear to be high, because NIGT1.1^L25A/L39A^ did not bind to a single copy of GAATC sequence strongly and competitor DNAs did not compete with labeled DNA probes effectively in EMSA (Fig 5C). These data suggest that dimerization of NIGT1 family proteins is involved in both the modulation of specificity and increase in affinity for target DNA sequences through interaction with a larger number of nucleotides.

Consistent with the observation that the NIGT1.1^WT^ protein exclusively forms a dimer in solution, dual mode DNA recognition allowed NIGT1 family proteins to recognize two types of palindromic sequences, GAATATTC and GATTC-N_38_-GAATC in the *NRT2*.*1* promoter, revealing the previously undetected recognition motifs. Some TFs, such as bacterial GabR and mammalian thyroid hormone receptors, bind as a dimer to *cis*-elements consisting of two sequences separated by a spacer [50–52]. Furthermore, binding of GabR TFs to the target DNA sequence is coupled with DNA bending because of the presence of a long (29 bp) spacer sequence. However, DNA binding of NIGT1 family proteins is distinguishable from that of GabR TFs. Binding of NIGT1 family proteins to the GATTC-N_38_-GAATC sequence needs to be coupled with a drastic conformational change in the NIGT1 dimer, because the orientation of the GAAT core sequence is opposite from those found in GAATATTC sequence. Repositioning of the DNA-binding domain, or existence of NIGT1 dimers in which the CCD of each monomer interacts in the opposite direction (i.e. parallel and antiparallel), as observed in CCDs of some proteins [53,54], may explain these phenomena. Furthermore, for binding to GATTC-N_38_-GAATC sequence, DNA bending might be necessary due to the presence of a large spacer sequence. Further studies are required to reveal precise mechanisms to provide new clues for resolving the confusing and/or seemingly conflicting results of DNA recognition by TFs.

### Dimerization of NIGT1 is critical for the proper control of nutrient responses

The results of the current study indicated that the dimerization of NIGT1 family proteins is important for binding to palindromic NIGT1-binding sites in the *SPX1* promoter and for modulating various aspects of PSR. The NIGT1 binding regions in the *NRT2*.*4* and *NRT2*.*5* gene promoters also contain the palindromic sequence for NIGT1 binding. These suggest that the dimerization ability of NIGT1 family proteins may have evolved to precisely bind to target gene promoters and to properly control nutrient responses.

The expression level of *SPX1* was inversely proportional to the amount of NIGT1.1 binding to the NIGT1 binding sites in the *SPX1* promoter. However, although the amount of NIGT1.1^L25A/L39A^ binding to the authentic NIGT1 site in the *NRT2*.*1* promoter was suggested to be smaller than that of NIGT1.1^WT^ binding to this site *in vivo*, NIGT1.1^WT^ and NIGT1.1^L25A/L39A^ suppressed *NRT2*.*1* expression to a similar extent *in planta* (Figs 4C and 6B), causing a remaining question. A possible explanation for this phenomenon is that binding of a small amount of NIGT1 family proteins to this site is sufficient to adequately suppress transcription in the context of the *NRT2*.*1* promoter *in planta*. Alternatively, NIGT1.1^L25A/L39A^ might artificially bind to GAATC motifs outside the region analyzed in ChIP assay *in vivo* and effectively repress *NRT2*.*1* expression. This unexpected observation suggests that the loss of dimerization of NIGT1 family proteins may generate complex effects on the gene expression profile *in planta*, further emphasizing the importance of dimerization-mediated precise binding of NIGT1 family proteins on nutrient responses.

Y2H assays revealed that NIGT1 family proteins are able to dimerize not only with other NIGT1 family proteins but also with some HHO family proteins. Nitrate-inducible genes encode NIGT1 family proteins, while non-nitrate-inducible genes encode HHO family proteins, suggesting that the physiological roles of HHO family proteins are different from those of NIGT family proteins. This is consistent with previous findings; HHO4 and HHO5 have been shown to modulate flowering time and meristem activity, respectively [55,56]. However, our recent results suggest that rice OsHHO3 and OsHHO4, close homologs of Arabidopsis HHO5 and HHO6, play central roles in N deficiency response in roots [10]. Considering that HHO5 and HHO6 interacted with only certain members of the NIGT1 family in this study, it is possible that homologous or heterologous dimer formation among NIGT1 and HHO family proteins is required for the regulation of some aspects of nutrient responses.

Although we focused on the role of the homologous NIGT1.1 dimer in the current study, formation of heterologous dimers comprising NIGT1 and HHO family proteins through interaction between the conserved CCDs may offer a more complex regulatory network for a variety of physiological processes. Untangling these complex networks will further our understanding of the mechanisms underlying nutrient signaling and will enable the design and implementation of desired nutrient responses.

## Materials and methods

### Plant materials and growth conditions

*Arabidopsis thaliana* ecotype Columbia-0 (Col-0) was used as the wild type in this study. The *nigtQ* quadruple mutant line was previously generated from four T-DNA insertion knockout single mutant lines, *nigt1*.*1* (GABI_267G03), *nigt1*.*2* (SALK_070096), *nigt1*.*3* (SAIL_28_D03), and *nigt1*.*4* (SALK_067074) [24,26], which were originally obtained from the Arabidopsis Biological Resource Center (ABRC), Ohio, USA [57]. Transgenic *nigtQ*/NIGT1.1^WT^ lines were generated previously [30].

To analyze gene expression and Pi concentration, seedlings were grown on vertically oriented agar plates containing modified half-strength Murashige and Skoog (1/2 MS) medium [58] supplemented with 0.5% sucrose, 3.5 mM MES (pH 5.7), and 0.8% agar. In the modified 1/2 MS medium, KH_2_PO_4_ concentration was changed to 0 or 500 μM to produce Pi-replete and Pi-deficient conditions, respectively, and iron (Fe) concentration was also reduced to 2 μM to avoid excess Fe under Pi-deficient conditions [59–61]. Seedlings were grown at 23°C under continuous illumination (60 μE m^-2^ s^-1^) for 9 d. For growth analysis using nitrate or ammonium as the sole N source, 5 mM KNO_3_ or 5 mM NH_4_Cl was added to N-free 1/2 MS medium. When ammonium was used as the sole N source, 5 mM KCl was additionally added to provide potassium ion. To measure the anthocyanin content, five seeds were cultured in 1.5 mL of modified 1/10 MS solution containing 0.5% sucrose, 3.5 mM MES (pH 5.7), and 10 or 1,000 μM KH_2_PO_4_ in a 24-well microtiter plate (#92424, TPP, Trasadingen, Switzerland). Seedlings were grown at 23°C under continuous light (60 μE m^-2^ s^-1^) for 9 d, with occasional shaking. For analysis of growth on the soil, seeds were first germinated on 1/2 MS agar plates, and 1-w-old seedlings were transferred to a nutrient-containing peat (Jiffy 7, Sakata Seed Corp., Yokohama, Japan). The plants were grown under continuous illumination (100 μE m^-2^ s^-1^) at 23°C for another 14 d before phenotypic analysis.

### Plasmid construction

To construct plasmids for Y2H assays, cDNAs of four *NIGT1* genes (*NIGT1*.*1*–*1*.*4*) were excised from *35S-C4PPDK-NIGT1–2×MYC* plasmids [24], and those of *HHO4–6* and *PHR1* were amplified from Col-0 mRNA by reverse transcription PCR (RT-PCR) using gene-specific primers (S1 Table). These cDNAs contained the coding region completely but did not 5' and 3'-untranslated regions, except for PHR1 cDNA that only contained part of the coding region corresponding to 208–362 aa. To synthesize mutant variants of the NIGT1.1 protein (NIGT1.1^L25A^, NIGT1.1^L39A^, or NIGT1.1^L25A/L39A^), mutations were introduced in the *NIGT1*.*1* cDNA by fusion PCR [62] using sequence-specific primers (S1 Table), and the mutated cDNAs were cloned into pGADT7 and pGBT9 plasmids (Takara Bio Inc., Kusatsu, Japan). To analyze the subcellular localization of NIGT1.1, the gene for ß-glucuronidase (GUS) in the pCB302-HYG-35S-GUS plasmid [11] was replaced by DNA fragments encoding NIGT1.1^WT^ or NIGT1.1^L25A/L39A^ fused in-frame to the N-terminus of GFP. To construct plasmids for BiFC assays, the *NIGT1*.*1* cDNA was first cloned into pENTR/D-TOPO (Thermo Fischer Scientific, Waltham, MA) and then introduced into Gateway-compatible BiFC vectors [63]. To create plasmids for production of recombinant Trx-6×His-NIGT1.1–6×His protein in *E*. *coli* (pET32-NIGT1.1 and pET32-NIGT1.1^L25A/L39A^), cDNAs encoding NIGT1.1^WT^ or NIGT1.1^L25A/L39A^ were cloned into pET32-b(+) (Merck Millipore, Burlington, MA) using *Nco*I and *Hin*dIII sites. A binary vector for generation of *nigtQ*/NIGT1.1^L25A/L39A^ lines was constructed by replacing the *NIGT1*.*1* cDNA in pCB302-HYG-UBQ10_pro_-NIGT1.1^WT^-6×MYC [24] with a DNA fragment encoding NIGT1.1^L25A/L39A^ protein. Plasmids for co-transfection assays in Arabidopsis seedlings (S4 Fig) were generated using the Gateway system using cDNAs encoding full-length, or the N-terminal or C-terminal region, of NIGT1.1, which was obtained by RT-PCR using Col-0 mRNA and sequence-specific primers (S1 Table). All PCR-generated inserts in the constructed plasmids were verified by DNA sequencing.

### Y2H assay

Y2H assays were conducted using yeast (*Saccharomyces cerevisiae*) strain AH109, as described previously [64]. After transformation of AH109 cells with the plasmids of interest, transformants were selected on synthetic defined (SD) medium lacking Leu and tryptophan (Trp; SD/-Leu/-Trp) at 30°C, and single colonies were streaked on plates containing SD/-Leu/-Trp or SD medium lacking Leu, Trp, His, and adenine (Ade; SD/-Leu/-Trp/-His/-Ade). Plates were photographed after 5 d of incubation at 30°C.

### Agroinfiltration and BiFC assay

*Agrobacterium tumefaciens* strain GV3101 (pMP90) was transformed with plasmids of interest and suspended in 0.2 mM acetosyringone. Leaves of 3-week-old *N*. *benthamiana* plants grown in soil were co-infiltrated, as described previously [65,66], with *Agrobacterium* carrying the plasmid of interest and *Agrobacterium* carrying the P19 silencing suppressor to suppress gene silencing [67,68]. Plasmids expressing the nGFP- or cGFP-GRC3 fusion [40] served as negative controls. GFP fluorescence derived from the introduced plasmids was observed using a fluorescence microscope (BX51; Olympus, Tokyo, Japan) equipped with a digital camera (DP80; Olympus) at 2 or 3 d post-agroinfiltration. Nuclei were stained with DAPI (Sigma, St. Louis, MO) infiltrated through the abaxial surface of the leaf prior to microscopy.

### Recombinant protein purification, SEC-MALS, and EMSA

*E*. *coli* strain Origami B (DE3) (Merck Millipore) was transformed with pET32-NIGT1.1 and pET32-NIGT1.1^L25A/L39A^ plasmids. Transformants cultured overnight in LB medium were inoculated into fresh LB medium and further cultured until the OD_600_ value reached 0.6. To induce recombinant protein production, the culture was treated with 1 mM isopropyl-β-D-thiogalactopyranoside (IPTG) at 15°C for 18 h. Soluble protein was extracted from the transformants by sonication in extraction buffer (50 mM sodium phosphate buffer [pH 8.0], 0.1% Triton X-100, 500 mM NaCl, and 1× EDTA-free protease inhibitor [#11873580001, Merck Millipore]) supplemented with 1 mM imidazole. Recombinant His-tagged protein in the extract was bound to TALON Metal Affinity Resin (#8901–1; Takara Bio Inc.), followed by two washes with the extraction buffer supplemented with 45 mM imidazole. Finally, Trx-6×His-NIGT1.1–6×His protein was eluted using extraction buffer supplemented with 250 mM imidazole. The eluted protein was mixed with an equal volume of glycerol and stored at -20°C until needed for further analysis.

SEC-MALS was carried out using Shodex PROTEIN LW-803 column (8.0 × 300 mm, Shoko Science Co., Ltd, Yokohama, Japan) connected to PROTEIN LW-G 6B guard column (6.0 × 50 mm, Shoko Science) in the buffer containing 20 mM Tris-HCl (pH 7.5), 500 mM NaCl and 10% glycerol at room temperature. Purified Trx-6×His-NIGT1.1^WT^-6×His and its mutant (Trx-6×His-NIGT1.1 ^L25A/L39A^ -6×His) (1 mg/ml, 70 μl) were loaded onto the column and then eluted with a flow rate of 0.8 ml min^–1^. Light scattering and absorbance at 280 nm were detected on a DAWN HELEOS II instrument (Wyatt Technology, Santa Barbara, CA) and an SPD-20A UV-VIS detector (Shimadzu Co. Kyoto, Japan), respectively. The light scattering detector was calibrated with bovine serum albumin before the measurements. Extinction coefficients at 280 nm, 0.826 ml mg^–1^ cm^–1^ (NIGT1.1^WT^) and 0.827 ml mg^–1^ cm^–1^ (NIGT1.1 ^L25A/L39A^), were used to calculate protein concentration. The data were analyzed with ASTRA software (Wyatt Technology).

EMSAs were performed using recombinant proteins as described previously [24]. DNA–protein binding was performed in a 15 μl reaction mixture containing 40 fmol of biotin-labeled probes in the presence or absence of molar excess of non-labeled competitor DNA, and electrophoresis was carried out using 4% polyacrylamide gels.

### ChIP assay

Wild-type, *nigtQ*/NIGT1.1^WT^ (#8), and *nigtQ*/NIGT1.1^L25A/L39A^ (#11) seedlings were grown on agar plates containing 1/2 MS medium, 0.5% (w/v) sucrose, 0.8% agar, and 3 mM MES-KOH (pH 5.8) for 12 days. Crosslinking of proteins to DNA using 1% (v/v) formaldehyde, isolation and lysis of nuclei, and DNA sonication using a Bioruptor II (Cosmo Bio, Tokyo, Japan) were carried out as described previously [24,69]. Anti-Myc polyclonal antibody (Clone 9E10, Millipore) and protein G-agarose beads (Merck Millipore) were used for immunoprecipitation. DNA recovered from agarose beads was purified using the DNeasy Plant Mini Kit (Qiagen, Hilden, Germany). qPCR was performed using the KAPA SYBR FAST qPCR kit (KAPA Biosystems) and gene-specific primers (S1 Table).

### Co-transfection assays using Arabidopsis seedlings and protoplasts

To analyze the repression domain of NIGT1 family proteins (S4 Fig), co-transfection assays were performed by particle bombardment using 10-d-old Arabidopsis seedlings and a particle delivery system (PDU-1000/He; Bio-Rad, Hercules, CA), as described previously [26]. The reporter plasmid contained the gene for firefly luciferase (FLUC) under the control of the GAL4-binding site-containing synthetic promoter [70], whereas effector plasmids were designed to express the fusion of GFP or NIGT1.1 with the GAL4 DNA-binding domain under the control of the cauliflower mosaic virus (CaMV) *35S* RNA promoter [26]. Additional effector plasmids expressing the N-terminal or C-terminal region of NIGT1.1 (1–48 and 49–344 aa, respectively) fused to the GAL4 DNA-binding domain were constructed using *NIGT1*.*1* cDNA fragments. The plasmid containing the gene for Renilla luciferase (RLUC) under the control of the CaMV *35S* RNA promoter served as an internal control [70]. Levels of FLUC and RLUC activity were measured using the Dual-Luciferase Reporter Assay System (Promega KK, Tokyo, Japan) using a microplate reader (Mithras LB940; Berthold, Bad Wildbad, Germany).

Co-transfection assays with Arabidopsis mesophyll protoplasts were performed as described previously [71]. Protoplasts were isolated from rosette leaves of 3-week-old wild-type Arabidopsis plants grown in 1/10 MS nutrient solution at 23°C under continuous illumination (60 μE m^-2^ s^-1^). The reporter plasmids containing FLUC gene under the control of the *SPX1* or *NRT2*.*1* promoter were reported previously [24,30], and effector plasmids were designed to express NIGT1.1^WT^-2×MYC or NIGT1.1^L25A/L39A^-2×MYC under the control of the constitutive *35S-C4PPDK* fusion promoter. A plasmid expressing 2×MYC under the control of the *35S-C4PPDK* promoter served as a control. Another plasmid expressing GUS gene under the control of the *UBIQUITIN10* (*UBQ10*) promoter was used as an internal control.

### Generation of transgenic Arabidopsis lines

To generate *nigtQ*/NIGT1.1^L25A//L39A^ lines, the *nigtQ* mutant was transformed with *A*. *tumefaciens* strain GV3101 (pMP90) carrying the pCB302-HYG-UBQ10_pro_-NIGT1.1^L25A/L39A^-6×MYC binary vector by the floral dip method [72]. Homozygous T3 or T4 seeds derived from T2 plants harboring the transgene at a single locus were used for subsequent experiments.

### Gene expression analysis

Total RNA extraction, cDNA synthesis, and quantitative real-time PCR (RT-qPCR) using gene-specific primers (S1 Table) were carried out as described previously [30]. Quantification of gene expression was based on the standard curve method using the *UBQ10* gene as an internal control.

### Measurement of Pi and anthocyanin contents

Foliar Pi concentration in 9-d-old seedlings was determined using the molybdenum blue method, as described previously [30,73]. Briefly, Pi was extracted from frozen pulverized samples using 1% acetic acid. Ten microliters of the extract was mixed with 80 μL of 0.35% (NH_4_)_2_MoO_4_ solution containing 0.5 M H_2_SO_4_ and 10 μL of 10% (w/v) ascorbic acid. After 1 h of incubation at 37°C, absorbance at 820 nm was recorded using a microplate reader (Infinite M1000, TECAN Ltd., Zürich, Switzerland). Serial dilutions of NaH_2_PO_4_ was prepared in 1% acetic acid and used to create a standard curve.

Anthocyanin content was determined using whole seedlings grown in liquid culture as described previously [30,40]. Briefly, five seedlings were immersed in 600 μL of 1% HCl prepared in methanol and shaken overnight at 4°C. Subsequently, 300 μL of the extract was mixed with 200 μL each of water and chloroform. After a brief centrifugation, the absorbance of the upper layer was recorded at 530 and 657 nm using a spectrophotometer. Relative anthocyanin content was calculated using the following equation:

$$Relative\;anthocyanin\;content=\frac{(A_{530}-0.25)\times A_{657}}{Sample\;FW}$$ (1)

where A_530_ and A_657_ represent the absorbance at 530 and 657 nm, respectively, and sample FW represents the fresh weight of whole seedlings (mg).

### Measurement of nitrate uptake

Arabidopsis seedlings grown on agar plates containing 1/2 MS medium, 0.5% (w/v) sucrose, 0.8% agar, and 3 mM MES-KOH (pH 5.8) for 10 days were used for the experiment. Nitrate uptake was measured using ^15^N-labeled nitrate, as described previously [24]. In brief, roots were submerged in 0.1 mM CaSO_4_ for 1 min, and then incubated in 0.2 mM K^15^NO_3_ for 5 min. After 1 min wash with 0.1 mM CaSO_4_, roots were completely dried. The N content and ^15^N/^14^N isotopic composition of roots were analyzed by SI Science Co., Ltd. (Yokohama, Japan).

### Statistical analysis

Two-tailed Student’s *t*-test was used to determine significant differences between wild type and *nigtQ* plants. To compare multiple genotypes including *nigtQ*/NIGT1.1^WT^ and *nigtQ*/NIGT1.1^L25A/L39A^ lines, one-way analysis of variance (ANOVA) was conducted, followed by Tukey’s Honest Significant Difference (HSD) post hoc test. In all comparisons, α = 0.05 was used as the significance threshold.

### Accession numbers

Sequence data from this article can be found in the Arabidopsis Genome Initiative under the following accession numbers: *NIGT1*.*1* (At1g25550), *NIGT1*.*2* (At1g68670), *NIGT1*.*3* (At3g25790), *NIGT1*.*4* (At1g13300), *HHO4* (At2g03500), *HHO5* (At4g37180), *HHO6* (At1g49560), *PHR1* (At4g28610), *GRC3* (At5g11010), *SPX1* (At5g20150), *NRT2*.*1* (At1g08090), *NRT2*.*4* (At5g60770), *NRT2*.*5* (At1g12940), *NAR2*.*1* (At5g50200), *UBQ10* (At4g05320), *PHT1;1* (At5g43350), *PHT1;4* (At2g38940), *IPS1* (At3g09922), and *PP2A* (At1g69960).

## Supporting information

S1 Fig Domain structure of Arabidopsis NIGT1 and HHO family proteins. The position of the coiled-coil domain (CCD) was predicted using the COILS website (https://embnet.vital-it.ch/software/COILS_form.html; accessed in 2018 June) [74]. The position of the GARP domain was determined using the Prosite database (https://prosite.expasy.org/; accessed in 2018 June) [75]. Predicted positions of the CCD and GARP domain are indicated in blue and red, respectively. Numbers indicate the amino acid positions. Phylogenetic tree showing the relationship among entire amino acid sequences was constructed with the MEGA7 software using the Neighbor-Joining method [76,77].
(DOCX)

S2 Fig Analysis of all pairwise combinations of proteins in yeast two-hybrid (Y2H) assays. Proteins fused to the GAL4 activation domain (AD) and DNA-binding domain (BD) are indicated. Truncated PHR1 (208–362 aa) was used as a negative control. Synthetic defined media (SD) lacking Leu and Trp (SD/-Leu/-Trp), or lacking Leu, Trp, His, and Ade (SD/-Leu/-Trp/-His/-Ade), was used.
(DOCX)

S3 Fig Quantification of *NIGT1*.*1* transcript and NIGT1.1 protein levels in *nigtQ*/NIGT1.1^WT^ and *nigtQ*/NIGT1.1^L25A/L39A^ plants. **(A)** *NIGT1*.*1* transcripts levels were analyzed in roots of 9-d-old seedlings grown on Pi-replete medium by RT-qPCR. Expression level in each transgenic line was normalized relative to that in the WT. Data represent mean ± SD (n = 4). **(B)** NIGT1.1 protein levels were analyzed in 7-d-old whole seedlings of *nigtQ*, *nigtQ*/NIGT1.1^WT^ and *nigtQ*/NIGT1.1^L25A/L39A^ lines grown with Pi-replete medium by western blotting using anti-MYC antibody. The Rubisco large subunit was detected by Coomassie Brilliant Blue (CBB) staining and shown as a loading control.
(DOCX)

S4 Fig The C-terminal half of NIGT1.1 is responsible for its transcriptional repressor activity. **(A**) Positions of the N- and C-terminal regions of NIGT1.1 (1–48 and 49–344 aa, respectively) used in the co-transfection assay in **(B)**. **(B)** Co-transfection assay. Reporter plasmid contained the firefly luciferase (FLUC) gene under the control of a GAL4-binding site-containing synthetic promoter. Each effector plasmid permitted the expression of full-length, N-terminal (1–48 aa), or C-terminal (49–344 aa) regions of NIGT1.1 fused in-frame to the GAL4 DNA-binding domain (BD). The reporter and effector plasmids were co-transfected into Arabidopsis leaves by particle bombardment. GFP fused to GAL4 BD was used as a control. FLUC activity was normalized relative to RLUC activity derived from an internal control plasmid [70], and the relative FLUC activity obtained using GAL4-GFP fusion was set to 1. Data represent mean ± standard deviation (SD; *n* = 4).
(DOCX)

S5 Fig Stability of NIGT1.1^WT^ and NIGT1.1^L25A/L39A^ proteins in seedlings. Four-day-old *nigtQ*/NIGT1.1^WT^ (#8) and *nigtQ*/NIGT1.1^L25A/L39A^ (#11) seedlings grown hydroponically with 1/2 MS medium were treated with 100 μM cycloheximide (+CHX, dissolved in DMSO) or DMSO alone (-CHX) for 9 h. Whole seeding was used for the analysis of protein level by western blotting using anti-MYC antibody. An electrophoresed band corresponding to Rubisco large subunit was stained with Coomassie Brilliant Blue (CBB) and shown as a loading control.
(DOCX)

S6 Fig Growth of *nigtQ*/NIGT1.1^WT^ and *nigtQ*/NIGT1.1^L25A/L39A^ seedlings on agar plates containing nitrate or ammonium as a sole N source. Phenotypes of WT, *nigtQ*, *nigtQ*/NIGT1.1^WT^, and *nigtQ*/NIGT1.1^L25A/L39A^ plants grown on 1/2 MS medium with 5 mM KNO_3_ **(A, B)** or 5 mM NH_4_Cl **(C, D)** as the sole nitrogen source. **(A, C)** Representative images of plants grown under respective condition. Scale bar = 4 mm. **(B, D)** Shoot fresh weight (FW) of plants grown under respective condition. Data represent mean ± SD (*n* = 17–21). In **(A)** and **(B)**, plants were grown for 9 d, and in **(C)** and **(D)**, plants were grown for 10 d.
(DOCX)

S7 Fig Growth and Pi content of soil-grown *nigtQ*/NIGT1.1^WT^ and *nigtQ*/NIGT1.1^L25A/L39A^ plants. **(A)** Phenotypes of WT, *nigtQ*, *nigtQ*/NIGT1.1^WT^, and *nigtQ*/NIGT1.1^L25A/L39A^ plants grown on 1/2 MS medium for 7 d and on soil for 14 d. Scale bars = 2 cm. **(B–D)** Box plots showing rosette leaf diameter **(B)**, shoot fresh weight (FW) **(C)**, and shoot Pi concentration **(D)** of plants grown under the same conditions as described in **(A)**. The middle horizontal line indicates the median value, and the upper and lower ends of each box indicate the upper and lower quantiles, respectively; *n* = 18 in **(B)**, 10 in **(C)**, and 9 in **(D)**. Significant differences between WT and *nigtQ* plants were determined using two-tailed Student’s *t*-test, and *P* values are indicated. Significant differences among *nigtQ*, *nigtQ*/NIGT1.1^WT^, and *nigtQ*/NIGT1.1^L25A/L39A^ lines were determined using one-way ANOVA, followed by Tukey’s HSD test, and are indicated using different lowercase letters.
(DOCX)

S8 Fig Variation in amino acid sequences of NIGT1 family proteins in Arabidopsis and rice. The frequency of amino acid variants and insertion/deletion mutations (indels) in Arabidopsis NIGT1 family proteins (NIGT1.1–1.4) and rice NIGT1 protein (OsNIGT1) is shown at each amino acid position. Sequences of NIGT1.1–1.4 were obtained from 1,135 naturally occurring Arabidopsis accessions and those of OsNIGT1.1 were obtained from 3,024 rice accessions. Only polymorphic sites are plotted, and a logarithmic scale is used for the y-axis. The CCD core (22 amino acids in Fig 1A) and the GARP domain in each protein are indicated with blue and red squares, respectively.
(DOCX)

S1 Table List of primers used in the study. (XLSX)

## References

[1] Bloom AJ, Chapin FS, Mooney HA. Resource limitation in plants-An economic analogy. Annu Rev Ecol Evol Syst. 1985;16:363–392.

[2] Richardson AE, Barea JM, McNeill AM, Prigent-Combaret C. Acquisition of phosphorus and nitrogen in the rhizosphere and plant growth promotion by microorganisms. Plant Soil. 2009;321:305–339.

[3] Kirk GJD, Kronzucker HJ. The potential for nitrification and nitrate uptake in the rhizosphere of wetland plants: A modelling study. Ann Bot. 2005;96:639–646. 10.1093/aob/mci216 16024557 PMC4247031

[4] Raghothama KG. Phosphate transport and signaling. Curr Opin Plant Biol. 2000;3:182–187. 10837272

[5] Bustos R, Castrillo G, Linhares F, Puga MI, Rubio V, Pérez-Pérez J, et al A central regulatory system largely controls transcriptional activation and repression responses to phosphate starvation in Arabidopsis. PLoS Genet. 2010;6:e1001102 10.1371/journal.pgen.1001102 20838596 PMC2936532

[6] Nilsson L, Müller R, Nielsen TH. Increased expression of the MYB-related transcription factor, *PHR1*, leads to enhanced phosphate uptake in *Arabidopsis thaliana*. Plant, Cell Environ. 2007;30:1499–1512. 10.1111/j.1365-3040.2007.01734.x 17927693

[7] Makino A. Photosynthesis, grain yield, and nitrogen utilization in rice and wheat. Plant Physiol. 2011;155:125–129. 10.1104/pp.110.165076 20959423 PMC3014227

[8] Vinod KK, Heuer S. Approaches towards nitrogen- and phosphorus-efficient rice. AoB Plants. 2012;2012: pls028 10.1093/aobpla/pls028 23115710 PMC3484362

[9] Xu G, Fan X, Miller AJ. Plant nitrogen assimilation and use efficiency. Annu Rev Plant Biol. 2012;63:153–182. 10.1146/annurev-arplant-042811-105532 22224450

[10] Ueda Y, Ohtsuki N, Kadota K, Tezuka A, Nagano AJ, Kadowaki T, et al Gene regulatory network and its constituent transcription factors that control nitrogen deficiency responses in rice. New Phytol. 2020;227:1434–1452. 10.1111/nph.16627 32343414

[11] Konishi M, Yanagisawa S. Arabidopsis NIN-like transcription factors have a central role in nitrate signalling. Nat Commun. 2013;4:1617 10.1038/ncomms2621 23511481

[12] Zhou J, Jiao F, Wu Z, Li Y, Wang X, He X, et al *OsPHR2* is involved in phosphate-starvation signaling and excessive phosphate accumulation in shoots of plants. Plant Physiol. 2008;146:1673–1686. 10.1104/pp.107.111443 18263782 PMC2287342

[13] Wang J, Sun J, Miao J, Guo J, Shi Z, He M, et al A phosphate starvation response regulator Ta-PHR1 is involved in phosphate signalling and increases grain yield in wheat. Ann Bot. 2013;111:1139–1153. 10.1093/aob/mct080 23589634 PMC3662521

[14] Rubio V, Linhares F, Solano R, Martín AC, Iglesias J, Leyva A, et al A conserved MYB transcription factor involved in phosphate starvation signaling both in vascular plants and in unicellular algae. Genes Dev. 2001;15:2122–2133. 11511543 10.1101/gad.204401 PMC312755

[15] Qi W, Manfield IW, Muench SP, Baker A. AtSPX1 affects the AtPHR1–DNA-binding equilibrium by binding monomeric AtPHR1 in solution. Biochem J. 2017;474:3675–3687. 10.1042/BCJ20170522 28887383 PMC5651819

[16] Osorio MB, Ng S, Berkowitz O, De Clercq I, Mao C, Shou H, et al SPX4 acts on PHR1-dependent and -independent regulation of shoot phosphorus status in Arabidopsis. Plant Physiol. 2019;181:332–352. 10.1104/pp.18.00594 31262954 PMC6716250

[17] Puga MI, Mateos I, Charukesi R, Wang Z, Franco-Zorrilla JM, De Lorenzo L, et al SPX1 is a phosphate-dependent inhibitor of PHOSPHATE STARVATION RESPONSE 1 in *Arabidopsis*. Proc Natl Acad Sci U S A. 2014;111:14947–14952. 10.1073/pnas.1404654111 25271326 PMC4205628

[18] Wild R, Gerasimaite R, Jung JY, Truffault V, Pavlovic I, Schmidt A, et al Control of eukaryotic phosphate homeostasis by inositol polyphosphate sensor domains. Science. 2016;352:986–990. 10.1126/science.aad9858 27080106

[19] Lv Q, Zhong Y, Wang Y, Wang Z, Zhang L, Shi J, et al SPX4 negatively regulates phosphate signaling and homeostasis through its interaction with PHR2 in rice. Plant Cell. 2014;26:1586–1597. 10.1105/tpc.114.123208 24692424 PMC4036573

[20] Nishida H, Tanaka S, Handa Y, Ito M, Sakamoto Y, Matsunaga S, et al A NIN-LIKE PROTEIN mediates nitrate-induced control of root nodule symbiosis in *Lotus japonicus*. Nat Commun. 2018;9:499. 29403008 10.1038/s41467-018-02831-x PMC5799372

[21] Wang M, Hasegawa T, Hayashi M, Ohmori Y, Yano K, Teramoto S, et al OsNLP4 is required for nitrate assimilation gene expressions and nitrate-dependent growth in rice. bioRxiv. 2020 10.1101/2020.03.16.993733

[22] Marchive C, Roudier F, Castaings L, Bréhaut V, Blondet E, Colot V, et al Nuclear retention of the transcription factor NLP7 orchestrates the early response to nitrate in plants. Nat Commun. 2013;4:1713 10.1038/ncomms2650 23591880

[23] Konishi M, Yanagisawa S. Emergence of a new step towards understanding the molecular mechanisms underlying nitrate-regulated gene expression. J Exp Bot. 2014;65:5589–5600. 10.1093/jxb/eru267 25005135

[24] Maeda Y, Konishi M, Kiba T, Sakuraba Y, Sawaki N, Kurai T, et al A NIGT1-centred transcriptional cascade regulates nitrate signalling and incorporates phosphorus starvation signals in *Arabidopsis*. Nat Commun. 2018;9:1376 10.1038/s41467-018-03832-6 29636481 PMC5893545

[25] Liu KH, Niu Y, Konishi M, Wu Y, Du H, Sun Chung H, et al Discovery of nitrate-CPK-NLP signalling in central nutrient-growth networks. Nature. 2017;545:311–316. 10.1038/nature22077 28489820 PMC5823009

[26] Kiba T, Inaba J, Kudo T, Ueda N, Konishi M, Mitsuda N, et al Repression of nitrogen starvation responses by members of the arabidopsis GARP-type transcription factor NIGT1/HRS1 subfamily. Plant Cell. 2018;30:925–945. 10.1105/tpc.17.00810 29622567 PMC5969275

[27] Sawaki N, Tsujimoto R, Shigyo M, Konishi M, Toki S, Fujiwara T, et al A nitrate-inducible GARP family gene encodes an auto-repressible transcriptional repressor in rice. Plant Cell Physiol. 2013;54:506–517. 10.1093/pcp/pct007 23324170

[28] Medici A, Marshall-Colon A, Ronzier E, Szponarski W, Wang R, Gojon A, et al AtNIGT1/HRS1 integrates nitrate and phosphate signals at the *Arabidopsis* root tip. Nat Commun. 2015;6:6274 10.1038/ncomms7274 25723764 PMC4373655

[29] Liu H, Yang H, Wu C, Feng J, Liu X, Qin H, et al Overexpressing HRS1 confers hypersensitivity to low phosphate-elicited inhibition of primary root growth in *Arabidopsis thaliana*. J Integr Plant Biol. 2009;51:382–392. 10.1111/j.1744-7909.2009.00819.x 19341407

[30] Ueda Y, Kiba T, Yanagisawa S. Nitrate-inducible NIGT1 proteins modulate phosphate uptake and starvation signalling via transcriptional regulation of *SPX* genes. Plant J. 2020;102:448–466. 10.1111/tpj.14637 31811679

[31] Ueda Y, Yanagisawa S. Perception, transduction, and integration of nitrogen and phosphorus nutritional signals in the transcriptional regulatory network in plants. J Exp Bot. 2019;70:3709–3717. 10.1093/jxb/erz148 30949701

[32] Yanagisawa S. Characterization of a nitrate-inducible transcriptional repressor NIGT1 provides new insights into DNA recognition by the GARP family proteins. Plant Signal Behav. 2013;8:e24447 10.4161/psb.24447 23603966 PMC3909032

[33] Hosoda K, Imamura A, Katoh E, Hatta T, Tachiki M, Yamada H, et al Molecular structure of the GARP family of plant Myb-related DNA binding motifs of the Arabidopsis response regulators. Plant Cell. 2002;14:2015–2029. 10.1105/tpc.002733 12215502 PMC150752

[34] Ruan W, Guo M, Xu L, Wang X, Zhao H, Wang J, et al An SPX-RLI1 module regulates leaf inclination in response to phosphate availability in rice. Plant Cell. 2018;30:853–870. 10.1105/tpc.17.00738 29610209 PMC5969273

[35] Waters MT, Wang P, Korkaric M, Capper RG, Saunders NJ, Langdale JA. GLK transcription factors coordinate expression of the photosynthetic apparatus in *Arabidopsis*. Plant Cell. 2009;21:1109–1128. 10.1105/tpc.108.065250 19376934 PMC2685620

[36] Safi A, Medici A, Szponarski W, Ruffel S, Lacombe B, Krouk G. The world according to GARP transcription factors. Curr Opin Plant Biol. 2017;39: 159–167. 10.1016/j.pbi.2017.07.006 28802165

[37] O’Shea EK, Rutkowski R, Kim PS. Evidence that the leucine zipper is a coiled coil. Science. 1989;243:538–542. 10.1126/science.2911757 2911757

[38] Landschulz WH, Johnson PF, McKnight SL. The leucine zipper: A hypothetical structure common to a new class of DNA binding proteins. Science. 1988;240:1759–1764. 10.1126/science.3289117 3289117

[39] Mason JM, Arndt KM. Coiled coil domains: Stability, specificity, and biological implications. ChemBioChem. 2004;5:170–176. 10.1002/cbic.200300781 14760737

[40] Maekawa S, Ishida T, Yanagisawa S. Reduced expression of APUM24, encoding a novel rRNA processing factor, induces sugar-dependent nucleolar stress and altered sugar responses in *Arabidopsis thaliana*. Plant Cell. 2018;30:209–227. 10.1105/tpc.17.00778 29242314 PMC5810573

[41] Das M, Kobayashi M, Yamada Y, Sreeramulu S, Ramakrishnan C, Wakatsuki S, et al Design of disulfide-linked thioredoxin dimers and multimers through analysis of crystal contacts. J Mol Biol. 2007;372:1278–1292. 10.1016/j.jmb.2007.07.033 17727880

[42] Shin H, Shin HS, Dewbre GR, Harrison MJ. Phosphate transport in *Arabidopsis*: Pht1;1 and Pht1;4 play a major role in phosphate acquisition from both low- and high-phosphate environments. Plant J. 2004;39:629–642. 10.1111/j.1365-313X.2004.02161.x 15272879

[43] Alonso-Blanco C, Andrade J, Becker C, Bemm F, Bergelson J, Borgwardt KMM, et al 1,135 genomes reveal the global pattern of polymorphism in *Arabidopsis thaliana*. Cell. 2016;166:481–491. 10.1016/j.cell.2016.05.063 27293186 PMC4949382

[44] Mansueto L, Fuentes RR, Borja FN, Detras J, Abrio-Santos JM, Chebotarov D, et al Rice SNP-seek database update: New SNPs, indels, and queries. Nucleic Acids Res. 2017;45:D1075–D1081. 10.1093/nar/gkw1135 27899667 PMC5210592

[45] Konishi M, Yanagisawa S. The role of protein-protein interactions mediated by the PB1 domain of NLP transcription factors in nitrate-inducible gene expression. BMC Plant Biol. 2019;19:90 10.1186/s12870-019-1692-3 30819094 PMC6393987

[46] Boer DR, Freire-Rios A, Van Den Berg WAM, Saaki T, Manfield IW, Kepinski S, et al Structural basis for DNA binding specificity by the auxin-dependent ARF transcription factors. Cell. 2014;156:577–589. 10.1016/j.cell.2013.12.027 24485461

[47] Chen WF, Wei X-B, Rety S, Huang LY, Liu NN, Dou SX, et al Structural analysis reveals a “molecular calipers” mechanism for a LATERAL ORGAN BOUNDARIES DOMAIN transcription factor protein from wheat. J Biol Chem. 2019;294:142–156. 10.1074/jbc.RA118.003956 30425099 PMC6322873

[48] Falvo J V., Lin CH, Tsytsykova A V., Hwang PK, Thanos D, Goldfeld AE, et al A dimer-specific function of the transcription factor NFATp. Proc Natl Acad Sci U S A. 2008;105: 19637–19642. 10.1073/pnas.0810648105 19060202 PMC2604938

[49] Amoutzias GD, Robertson DL, Van de Peer Y, Oliver SG. Choose your partners: dimerization in eukaryotic transcription factors. Trends Biochem Sci. 2008;33:220–229. 10.1016/j.tibs.2008.02.002 18406148

[50] Umesono K, Murakami KK, Thompson CC, Evans RM. Direct repeats as selective response elements for the thyroid hormone, retinoic acid, and vitamin D3 receptors. Cell. 1991;65:1255–1266. 10.1016/0092-8674(91)90020-y 1648450 PMC6159884

[51] Zechel C, Shen XQ, Chambon P, Gronemeyer H. Dimerization interfaces formed between the DNA binding domains determine the cooperative binding of RXR/RAR and RXR/TR heterodimers to DR5 and DR4 elements. EMBO J. 1994;13:1414–1424. 8137825 10.1002/j.1460-2075.1994.tb06395.x PMC394959

[52] Al-Zyoud WA, Hynson RMG, Ganuelas LA, Coster ACF, Duff AP, Baker MAB, et al Binding of transcription factor GabR to DNA requires recognition of DNA shape at a location distinct from its cognate binding site. Nucleic Acids Res. 2016;44:1411–1420. 10.1093/nar/gkv1466 26681693 PMC4756830

[53] Oakley MG, Kim PS. A buried polar interaction can direct the relative orientation of helices in a coiled coil. Biochemistry. 1998;37:12603–12610. 10.1021/bi981269m 9730833

[54] Kapinos LE, Burkhard P, Herrmann H, Aebi U, Strelkov S V. Simultaneous formation of right- and left-handed anti-parallel coiled-coil interfaces by a Coil2 fragment of human lamin A. J Mol Biol. 2011;408:135–146. 10.1016/j.jmb.2011.02.037 21354179

[55] Yan Y, Shen L, Chen Y, Bao S, Thong Z, Yu H. A MYB-domain protein EFM mediates flowering responses to environmental cues in *Arabidopsis*. Dev Cell. 2014;30:437–448. 10.1016/j.devcel.2014.07.004 25132385

[56] Moreau F, Thévenon E, Blanvillain R, Lopez-Vidriero I, Franco-Zorrilla JM, Dumas R, et al The Myb-domain protein ULTRAPETALA1 INTERACTING FACTOR 1 controls floral meristem activities in Arabidopsis. Dev. 2016;143:1108–1119. 10.1242/dev.127365 26903506

[57] Alonso JM, Stepanova AN, Leisse TJ, Kim CJ, Chen H, Shinn P, et al Genome-wide insertional mutagenesis of *Arabidopsis thaliana*. Science. 2003;301:653–657. 10.1126/science.1086391 12893945

[58] Murashige T, Skoog F. A revised medium for rapid growth and bio assays with Tobacco tissue cultures. Physiol Plant. 1962;15:474–497.

[59] Ward JT, Lahner B, Yakubova E, Salt DE, Raghothama KG. The effect of iron on the primary root elongation of Arabidopsis during phosphate deficiency. Plant Physiol. 2008;147:1181–1191. 10.1104/pp.108.118562 18467463 PMC2442553

[60] Mora-Macías J, Ojeda-Rivera JO, Gutiérrez-Alanís D, Yong-Villalobos L, Oropeza-Aburto A, Raya-González J, et al Malate-dependent Fe accumulation is a critical checkpoint in the root developmental response to low phosphate. Proc Natl Acad Sci U S A. 2017;114:E3563–E3572. 10.1073/pnas.1701952114 28400510 PMC5410833

[61] Balzergue C, Dartevelle T, Godon C, Laugier E, Meisrimler C, Teulon JM, et al Low phosphate activates STOP1-ALMT1 to rapidly inhibit root cell elongation. Nat Commun. 2017;8:15300 10.1038/ncomms15300 28504266 PMC5440667

[62] Szewczyk E, Nayak T, Oakley CE, Edgerton H, Xiong Y, Taheri-Talesh N, et al Fusion PCR and gene targeting in *Aspergillus nidulans*. Nat Protoc. 2006;1:3111–3120. 10.1038/nprot.2006.405 17406574

[63] Tanaka Y, Kimura T, Hikino K, Goto S, Nishimura M, Mano S, et al Gateway vectors for plant genetic engineering: overview of plant vectors, application for bimolecular fluorescence complementation (BiFC) and multigene construction In: Barrera-Saldaña HA, editor. Genetic Engineering—Basics, New Applications and Responsibilities. Rijeka: InTechOpen; 2012.

[64] Gietz RD, Schiestl RH. Frozen competent yeast cells that can be transformed with high efficiency using the LiAc/SS carrier DNA/PEG method. Nat Protoc. 2007;2:1–4. 10.1038/nprot.2007.17 17401330

[65] Sparkes IA, Runions J, Kearns A, Hawes C. Rapid, transient expression of fluorescent fusion proteins in tobacco plants and generation of stably transformed plants. Nat Protoc. 2006;1:2019–25. 10.1038/nprot.2006.286 17487191

[66] Ueda Y, Siddique S, Frei M. A novel gene, *OZONE-RESPONSIVE APOPLASTIC PROTEIN1*, enhances cell death in ozone stress in rice. Plant Physiol. 2015;169:873–889. 10.1104/pp.15.00956 26220952 PMC4577431

[67] Shah KH, Almaghrabi B, Bohlmann H. Comparison of expression vectors for transient expression of recombinant proteins in plants. Plant Mol Biol Report. 2013;31:1529–1538. 10.1007/s11105-013-0614-z 24415845 PMC3881577

[68] Liu L, Zhang Y, Tang S, Zhao Q, Zhang Z, Zhang H, et al An efficient system to detect protein ubiquitination by agroinfiltration in *Nicotiana benthamiana*. Plant J. 2010;61:893–903. 10.1111/j.1365-313X.2009.04109.x 20015064

[69] Saleh A, Alvarez-Venegas R, Avramova Z. An efficient chromatin immunoprecipitation (ChIP) protocol for studying histone modifications in *Arabidopsis* plants. Nat Protoc. 2008;3:1018–1025. 10.1038/nprot.2008.66 18536649

[70] Fujimoto SY, Ohta M, Usui A, Shinshi H, Ohme-Takagi M. Arabidopsis ethylene-responsive element binding factors act as transcriptional activators or repressors of GCC box-mediated gene expression. Plant Cell. 2000;12:393–404. 10.1105/tpc.12.3.393 10715325 PMC139839

[71] Yoo S-D, Cho Y-H, Sheen J. *Arabidopsis* mesophyll protoplasts: a versatile cell system for transient gene expression analysis. Nat Protoc. 2007;2:1565–1572. 10.1038/nprot.2007.199 17585298

[72] Clough SJ, Bent AF. Floral dip: a simplified method for Agrobacterium-mediated transformation of *Arabidopsis thaliana*. Plant J. 1998;16:735–743. 10069079 10.1046/j.1365-313x.1998.00343.x

[73] Kanno S, Cuyas L, Javot H, Bligny R, Gout E, Dartevelle T, et al Performance and limitations of phosphate quantification: guidelines for plant biologists. Plant Cell Physiol. 2016;57:690–706. 10.1093/pcp/pcv208 26865660

[74] Lupas A, Van Dyke M, Stock J. Predicting coiled coils from protein sequences. Science. 1991;252:1162–1164. 10.1126/science.252.5009.1162 2031185

[75] de Castro E, Sigrist CJA, Gattiker A, Bulliard V, Langendijk-Genevaux PS, Gasteiger E, et al ScanProsite: detection of PROSITE signature matches and ProRule-associated functional and structural residues in proteins. Nucleic Acids Res. 2006;34:W362–W365. 10.1093/nar/gkl124 16845026 PMC1538847

[76] Saitou N, Nei M. The neighbor-joining method: a new method for reconstructing phylogenetic trees. Mol Biol Evol. 1987;4:406–425 10.1093/oxfordjournals.molbev.a040454 3447015

[77] Kumar S, Stecher G, Tamura K. MEGA7: Molecular Evolutionary Genetics Analysis version 7.0 for bigger datasets. Mol Biol Evol. 2016;33:1870–1874. 10.1093/molbev/msw054 27004904 PMC8210823

