# Supplementary material for: NIGT1 family proteins exhibit dual mode DNA recognition to regulate nutrient response-associated genes in Arabidopsis
Source: PLoS Genet. 2020 Nov 2;16(11):e1009197. doi: 10.1371/journal.pgen.1009197 (PMC7660924; doi:10.1371/journal.pgen.1009197)
Supplement: S1 Fig — The position of the coiled-coil domain (CCD) was predicted using the COILS website (https://embnet.vital-it.ch/software/COILS_form.html; accessed in 2018 June) [74]. The position of the GARP domain was determined using the Prosite database (https://prosite.expasy.org/; accessed in 2018 June) [75]. Predicted positions of the CCD and GARP domain are indicated in blue and red, respectively. Numbers indicate the amino acid positions. Phylogenetic tree showing the relationship among entire amino acid sequences was constructed with the MEGA7 software using the Neighbor-Joining method [76,77]. (DOCX) [file pgen.1009197.s001.docx]

**S1 Fig| Domain structure of Arabidopsis NIGT1 and HHO family proteins.**

The position of the coiled-coil domain (CCD) was predicted using the COILS website (https://embnet.vital-it.ch/software/COILS_form.html; accessed in 2018 June) [74]. The position of the GARP domain was determined using the Prosite database (https://prosite.expasy.org/; accessed in 2018 June) [75]. Predicted positions of the CCD and GARP domain are indicated in blue and red, respectively. Numbers indicate the amino acid positions. Phylogenetic tree showing the relationship among entire amino acid sequences was constructed with the MEGA7 software using the Neighbor-Joining method [76,77].
