# Supplementary material for: NIGT1 family proteins exhibit dual mode DNA recognition to regulate nutrient response-associated genes in Arabidopsis
Source: PLoS Genet. 2020 Nov 2;16(11):e1009197. doi: 10.1371/journal.pgen.1009197 (PMC7660924; doi:10.1371/journal.pgen.1009197)
Supplement: S2 Fig — Proteins fused to the GAL4 activation domain (AD) and DNA-binding domain (BD) are indicated. Truncated PHR1 (208–362 aa) was used as a negative control. Synthetic defined media (SD) lacking Leu and Trp (SD/-Leu/-Trp), or lacking Leu, Trp, His, and Ade (SD/-Leu/-Trp/-His/-Ade), was used. (DOCX) [file pgen.1009197.s002.docx]

**S2 Fig| Analysis of all pairwise combinations of proteins in yeast two-hybrid (Y2H) assays.**

Proteins fused to the GAL4 activation domain (AD) and DNA-binding domain (BD) are indicated. Truncated PHR1 (208–362 aa) was used as a negative control. Synthetic defined media (SD) lacking Leu and Trp (SD/-Leu/-Trp), or lacking Leu, Trp, His, and Ade (SD/-Leu/-Trp/-His/-Ade), was used.
