# Supplementary material for: NIGT1 family proteins exhibit dual mode DNA recognition to regulate nutrient response-associated genes in Arabidopsis
Source: PLoS Genet. 2020 Nov 2;16(11):e1009197. doi: 10.1371/journal.pgen.1009197 (PMC7660924; doi:10.1371/journal.pgen.1009197)
Supplement: S3 Fig — (A) NIGT1.1 transcripts levels were analyzed in roots of 9-d-old seedlings grown on Pi-replete medium by RT-qPCR. Expression level in each transgenic line was normalized relative to that in the WT. Data represent mean ± SD (n = 4). (B) NIGT1.1 protein levels were analyzed in 7-d-old whole seedlings of nigtQ, nigtQ/NIGT1.1WT and nigtQ/NIGT1.1L25A/L39A lines grown with Pi-replete medium by western blotting using anti-MYC antibody. The Rubisco large subunit was detected by Coomassie Brilliant Blue (CBB) staining and shown as a loading control. (DOCX) [file pgen.1009197.s003.docx]

**S3 Fig| Quantification of *NIGT1.1* transcript and NIGT1.1 protein levels in *nigtQ*/NIGT1.1^WT^ and *nigtQ*/NIGT1.1^L25A/L39A^ plants.**

**(A)** *NIGT1.1* transcripts levels were analyzed in roots of 9-d-old seedlings grown on Pi-replete medium by RT-qPCR. Expression level in each transgenic line was normalized relative to that in the WT. Data represent mean ± SD (n = 4).

**(B)** NIGT1.1 protein levels were analyzed in 7-d-old whole seedlings of *nigtQ*, *nigtQ*/NIGT1.1^WT^ and *nigtQ*/NIGT1.1^L25A/L39A^ lines grown with Pi-replete medium by western blotting using anti-MYC antibody. The Rubisco large subunit was detected by Coomassie Brilliant Blue (CBB) staining and shown as a loading control.
