# Supplementary material for: NIGT1 family proteins exhibit dual mode DNA recognition to regulate nutrient response-associated genes in Arabidopsis
Source: PLoS Genet. 2020 Nov 2;16(11):e1009197. doi: 10.1371/journal.pgen.1009197 (PMC7660924; doi:10.1371/journal.pgen.1009197)
Supplement: S4 Fig — (A) Positions of the N- and C-terminal regions of NIGT1.1 (1–48 and 49–344 aa, respectively) used in the co-transfection assay in (B). (B) Co-transfection assay. Reporter plasmid contained the firefly luciferase (FLUC) gene under the control of a GAL4-binding site-containing synthetic promoter. Each effector plasmid permitted the expression of full-length, N-terminal (1–48 aa), or C-terminal (49–344 aa) regions of NIGT1.1 fused in-frame to the GAL4 DNA-binding domain (BD). The reporter and effector plasmids were co-transfected into Arabidopsis leaves by particle bombardment. GFP fused to GAL4 BD was used as a control. FLUC activity was normalized relative to RLUC activity derived from an internal control plasmid [70], and the relative FLUC activity obtained using GAL4-GFP fusion was set to 1. Data represent mean ± standard deviation (SD; n = 4). (DOCX) [file pgen.1009197.s004.docx]

**S4 Fig| The C-terminal half of NIGT1.1 is responsible for its transcriptional repressor activity.**

**(A**) Positions of the N- and C-terminal regions of NIGT1.1 (1–48 and 49–344 aa, respectively) used in the co-transfection assay in **(B)**.

**(B)** Co-transfection assay. Reporter plasmid contained the firefly luciferase (FLUC) gene under the control of a GAL4-binding site-containing synthetic promoter. Each effector plasmid permitted the expression of full-length, N-terminal (1–48 aa), or C-terminal (49–344 aa) regions of NIGT1.1 fused in-frame to the GAL4 DNA-binding domain (BD). The reporter and effector plasmids were co-transfected into Arabidopsis leaves by particle bombardment. GFP fused to GAL4 BD was used as a control. FLUC activity was normalized relative to RLUC activity derived from an internal control plasmid [70], and the relative FLUC activity obtained using GAL4-GFP fusion was set to 1. Data represent mean ± standard deviation (SD; *n* = 4).

**S5 Fig| Stability of NIGT1.1^WT^ and NIGT1.1^L25A/L39A^ proteins in seedlings.**

Four-day-old *nigtQ*/NIGT1.1^WT^ (#8) and *nigtQ*/NIGT1.1^L25A/L39A^ (#11) seedlings grown hydroponically with 1/2 MS medium were treated with 100 μM cycloheximide (+CHX, dissolved in DMSO) or DMSO alone (-CHX) for 9 h. Whole seeding was used for the analysis of protein level by western blotting using anti-MYC antibody. An electrophoresed band corresponding to Rubisco large subunit was stained with Coomassie Brilliant Blue (CBB) and shown as a loading control.

**S6 Fig| Growth of *nigtQ*/NIGT1.1^WT^ and *nigtQ*/NIGT1.1^L25A/L39A^ seedlings on agar plates containing nitrate or ammonium as a sole N source.**

Phenotypes of WT, *nigtQ*, *nigtQ*/NIGT1.1^WT^, and *nigtQ*/NIGT1.1^L25A/L39A^ plants grown on 1/2 MS medium with 5 mM KNO_3_ **(A, B)** or 5 mM NH_4_Cl **(C, D)** as the sole nitrogen source.

**(A, C)** Representative images of plants grown under respective condition. Scale bar = 4 mm. **(B, D)** Shoot fresh weight (FW) of plants grown under respective condition. Data represent mean ± SD (*n* = 17-21). In **(A)** and **(B)**, plants were grown for 9 d, and in **(C)** and **(D)**, plants were grown for 10 d.

**S7 Fig| Growth and Pi content of soil-grown *nigtQ*/NIGT1.1^WT^ and *nigtQ*/NIGT1.1^L25A/L39A^ plants.**

**(A)** Phenotypes of WT, *nigtQ*, *nigtQ*/NIGT1.1^WT^, and *nigtQ*/NIGT1.1^L25A/L39A^ plants grown on 1/2 MS medium for 7 d and on soil for 14 d. Scale bars = 2 cm.

**(B–D)** Box plots showing rosette leaf diameter **(B)**, shoot fresh weight (FW) **(C)**, and shoot Pi concentration **(D)** of plants grown under the same conditions as described in **(A)**. The middle horizontal line indicates the median value, and the upper and lower ends of each box indicate the upper and lower quantiles, respectively; *n* = 18 in **(B)**, 10 in **(C)**, and 9 in **(D)**. Significant differences between WT and *nigtQ* plants were determined using two-tailed Student’s *t*-test, and *P* values are indicated. Significant differences among *nigtQ*, *nigtQ*/NIGT1.1^WT^, and *nigtQ*/NIGT1.1^L25A/L39A^ lines were determined using one-way ANOVA, followed by Tukey’s HSD test, and are indicated using different lowercase letters.

**S8 Fig| Variation in amino acid sequences of NIGT1 family proteins in Arabidopsis and rice.**

The frequency of amino acid variants and insertion/deletion mutations (indels) in Arabidopsis NIGT1 family proteins (NIGT1.1–1.4) and rice NIGT1 protein (OsNIGT1) is shown at each amino acid position. Sequences of NIGT1.1–1.4 were obtained from 1,135 naturally occurring Arabidopsis accessions and those of OsNIGT1.1 were obtained from 3,024 rice accessions. Only polymorphic sites are plotted, and a logarithmic scale is used for the y-axis. The CCD core (22 amino acids in Fig 1A) and the GARP domain in each protein are indicated with blue and red squares, respectively.
