# Supplementary material for: NIGT1 family proteins exhibit dual mode DNA recognition to regulate nutrient response-associated genes in Arabidopsis
Source: PLoS Genet. 2020 Nov 2;16(11):e1009197. doi: 10.1371/journal.pgen.1009197 (PMC7660924; doi:10.1371/journal.pgen.1009197)
Supplement: S5 Fig — Four-day-old nigtQ/NIGT1.1WT (#8) and nigtQ/NIGT1.1L25A/L39A (#11) seedlings grown hydroponically with 1/2 MS medium were treated with 100 μM cycloheximide (+CHX, dissolved in DMSO) or DMSO alone (-CHX) for 9 h. Whole seeding was used for the analysis of protein level by western blotting using anti-MYC antibody. An electrophoresed band corresponding to Rubisco large subunit was stained with Coomassie Brilliant Blue (CBB) and shown as a loading control. (DOCX) [file pgen.1009197.s005.docx]

**S5 Fig| Stability of NIGT1.1^WT^ and NIGT1.1^L25A/L39A^ proteins in seedlings.**

Four-day-old *nigtQ*/NIGT1.1^WT^ (#8) and *nigtQ*/NIGT1.1^L25A/L39A^ (#11) seedlings grown hydroponically with 1/2 MS medium were treated with 100 μM cycloheximide (+CHX, dissolved in DMSO) or DMSO alone (-CHX) for 9 h. Whole seeding was used for the analysis of protein level by western blotting using anti-MYC antibody. An electrophoresed band corresponding to Rubisco large subunit was stained with Coomassie Brilliant Blue (CBB) and shown as a loading control.
