# Supplementary material for: NIGT1 family proteins exhibit dual mode DNA recognition to regulate nutrient response-associated genes in Arabidopsis
Source: PLoS Genet. 2020 Nov 2;16(11):e1009197. doi: 10.1371/journal.pgen.1009197 (PMC7660924; doi:10.1371/journal.pgen.1009197)
Supplement: S6 Fig — Phenotypes of WT, nigtQ, nigtQ/NIGT1.1WT, and nigtQ/NIGT1.1L25A/L39A plants grown on 1/2 MS medium with 5 mM KNO3 (A, B) or 5 mM NH4Cl (C, D) as the sole nitrogen source. (A, C) Representative images of plants grown under respective condition. Scale bar = 4 mm. (B, D) Shoot fresh weight (FW) of plants grown under respective condition. Data represent mean ± SD (n = 17–21). In (A) and (B), plants were grown for 9 d, and in (C) and (D), plants were grown for 10 d. (DOCX) [file pgen.1009197.s006.docx]

**S6 Fig| Growth of *nigtQ*/NIGT1.1^WT^ and *nigtQ*/NIGT1.1^L25A/L39A^ seedlings on agar plates containing nitrate or ammonium as a sole N source.**

Phenotypes of WT, *nigtQ*, *nigtQ*/NIGT1.1^WT^, and *nigtQ*/NIGT1.1^L25A/L39A^ plants grown on 1/2 MS medium with 5 mM KNO_3_ **(A, B)** or 5 mM NH_4_Cl **(C, D)** as the sole nitrogen source.

**(A, C)** Representative images of plants grown under respective condition. Scale bar = 4 mm. **(B, D)** Shoot fresh weight (FW) of plants grown under respective condition. Data represent mean ± SD (*n* = 17-21). In **(A)** and **(B)**, plants were grown for 9 d, and in **(C)** and **(D)**, plants were grown for 10 d.
