# Supplementary material for: NIGT1 family proteins exhibit dual mode DNA recognition to regulate nutrient response-associated genes in Arabidopsis
Source: PLoS Genet. 2020 Nov 2;16(11):e1009197. doi: 10.1371/journal.pgen.1009197 (PMC7660924; doi:10.1371/journal.pgen.1009197)
Supplement: S7 Fig — (A) Phenotypes of WT, nigtQ, nigtQ/NIGT1.1WT, and nigtQ/NIGT1.1L25A/L39A plants grown on 1/2 MS medium for 7 d and on soil for 14 d. Scale bars = 2 cm. (B–D) Box plots showing rosette leaf diameter (B), shoot fresh weight (FW) (C), and shoot Pi concentration (D) of plants grown under the same conditions as described in (A). The middle horizontal line indicates the median value, and the upper and lower ends of each box indicate the upper and lower quantiles, respectively; n = 18 in (B), 10 in (C), and 9 in (D). Significant differences between WT and nigtQ plants were determined using two-tailed Student’s t-test, and P values are indicated. Significant differences among nigtQ, nigtQ/NIGT1.1WT, and nigtQ/NIGT1.1L25A/L39A lines were determined using one-way ANOVA, followed by Tukey’s HSD test, and are indicated using different lowercase letters. (DOCX) [file pgen.1009197.s007.docx]

**S7 Fig| Growth and Pi content of soil-grown *nigtQ*/NIGT1.1^WT^ and *nigtQ*/NIGT1.1^L25A/L39A^ plants.**

**(A)** Phenotypes of WT, *nigtQ*, *nigtQ*/NIGT1.1^WT^, and *nigtQ*/NIGT1.1^L25A/L39A^ plants grown on 1/2 MS medium for 7 d and on soil for 14 d. Scale bars = 2 cm.

**(B–D)** Box plots showing rosette leaf diameter **(B)**, shoot fresh weight (FW) **(C)**, and shoot Pi concentration **(D)** of plants grown under the same conditions as described in **(A)**. The middle horizontal line indicates the median value, and the upper and lower ends of each box indicate the upper and lower quantiles, respectively; *n* = 18 in **(B)**, 10 in **(C)**, and 9 in **(D)**. Significant differences between WT and *nigtQ* plants were determined using two-tailed Student’s *t*-test, and *P* values are indicated. Significant differences among *nigtQ*, *nigtQ*/NIGT1.1^WT^, and *nigtQ*/NIGT1.1^L25A/L39A^ lines were determined using one-way ANOVA, followed by Tukey’s HSD test, and are indicated using different lowercase letters.
