# Supplementary material for: NIGT1 family proteins exhibit dual mode DNA recognition to regulate nutrient response-associated genes in Arabidopsis
Source: PLoS Genet. 2020 Nov 2;16(11):e1009197. doi: 10.1371/journal.pgen.1009197 (PMC7660924; doi:10.1371/journal.pgen.1009197)
Supplement: S8 Fig — The frequency of amino acid variants and insertion/deletion mutations (indels) in Arabidopsis NIGT1 family proteins (NIGT1.1–1.4) and rice NIGT1 protein (OsNIGT1) is shown at each amino acid position. Sequences of NIGT1.1–1.4 were obtained from 1,135 naturally occurring Arabidopsis accessions and those of OsNIGT1.1 were obtained from 3,024 rice accessions. Only polymorphic sites are plotted, and a logarithmic scale is used for the y-axis. The CCD core (22 amino acids in Fig 1A) and the GARP domain in each protein are indicated with blue and red squares, respectively. (DOCX) [file pgen.1009197.s008.docx]

**S8 Fig| Variation in amino acid sequences of NIGT1 family proteins in Arabidopsis and rice.**

The frequency of amino acid variants and insertion/deletion mutations (indels) in Arabidopsis NIGT1 family proteins (NIGT1.1–1.4) and rice NIGT1 protein (OsNIGT1) is shown at each amino acid position. Sequences of NIGT1.1–1.4 were obtained from 1,135 naturally occurring Arabidopsis accessions and those of OsNIGT1.1 were obtained from 3,024 rice accessions. Only polymorphic sites are plotted, and a logarithmic scale is used for the y-axis. The CCD core (22 amino acids in Fig 1A) and the GARP domain in each protein are indicated with blue and red squares, respectively.
